# RNA polymerase redistribution supports growth in *E. coli* strains with a minimal number of rRNA operons

**DOI:** 10.1093/nar/gkad511

**Published:** 2023-06-23

**Authors:** Jun Fan, Hafez El Sayyed, Oliver J Pambos, Mathew Stracy, Jingwen Kyropoulos, Achillefs N Kapanidis

**Affiliations:** Biological Physics Research Group, Clarendon Laboratory, Department of Physics, University of Oxford, Oxford OX1 3PU, UK; Institute of Fundamental and Frontier Sciences, University of Electronic Science and Technology of China, Chengdu, Sichuan 611731, China; Biological Physics Research Group, Clarendon Laboratory, Department of Physics, University of Oxford, Oxford OX1 3PU, UK; Kavli Institute for Nanoscience Discovery, Dorothy Crowfoot Hodgkin building, University of Oxford, Sherrington Road, Oxford OX1 3QU, UK; Biological Physics Research Group, Clarendon Laboratory, Department of Physics, University of Oxford, Oxford OX1 3PU, UK; Kavli Institute for Nanoscience Discovery, Dorothy Crowfoot Hodgkin building, University of Oxford, Sherrington Road, Oxford OX1 3QU, UK; Biological Physics Research Group, Clarendon Laboratory, Department of Physics, University of Oxford, Oxford OX1 3PU, UK; Department of Biochemistry, University of Oxford, Oxford OX1 3QU, UK; Biological Physics Research Group, Clarendon Laboratory, Department of Physics, University of Oxford, Oxford OX1 3PU, UK; Biological Physics Research Group, Clarendon Laboratory, Department of Physics, University of Oxford, Oxford OX1 3PU, UK; Kavli Institute for Nanoscience Discovery, Dorothy Crowfoot Hodgkin building, University of Oxford, Sherrington Road, Oxford OX1 3QU, UK

## Abstract

Bacterial transcription by RNA polymerase (RNAP) is spatially organized. RNAPs transcribing highly expressed genes locate in the nucleoid periphery, and form clusters in rich medium, with several studies linking RNAP clustering and transcription of rRNA (*rrn*). However, the nature of RNAP clusters and their association with *rrn* transcription remains unclear. Here we address these questions by using single-molecule tracking to monitor the subcellular distribution of mobile and immobile RNAP in strains with a heavily reduced number of chromosomal *rrn* operons (*Δrrn* strains). Strikingly, we find that the fraction of chromosome-associated RNAP (which is mainly engaged in transcription) is robust to deleting five or six of the seven chromosomal *rrn* operons. Spatial analysis in *Δrrn* strains showed substantial RNAP redistribution during moderate growth, with clustering increasing at cell endcaps, where the remaining *rrn* operons reside. These results support a model where RNAPs in *Δrrn* strains relocate to copies of the remaining *rrn* operons. In rich medium, *Δrrn* strains redistribute RNAP to minimize growth defects due to *rrn* deletions, with very high RNAP densities on *rrn* genes leading to genomic instability. Our study links RNAP clusters and *rrn* transcription, and offers insight into how bacteria maintain growth in the presence of only 1–2 *rrn* operons.

## Graphical Abstract

**Graphical Abstract:**
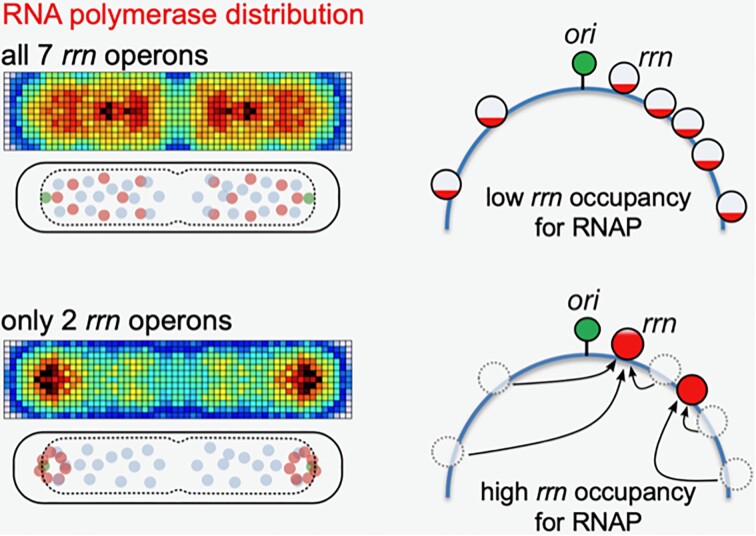

## INTRODUCTION

Transcription, a central process in gene expression, is spatially organized in many organisms; this organization is thought to increase the efficiency for RNA synthesis (1) and help cells adapt to different growth environments, nutrients and types of stress. In eukaryotes, synthesis of rRNA by RNA polymerase (RNAP) I occurs in the nucleolus, a nuclear compartment (2); further, eukaryotic mRNA transcription occurs in spatially enriched foci called ‘transcription factories’ (3), which contain RNAP II clusters (4) with lifetimes correlated to the levels of mRNA synthesis (5). Some viral transcription systems are also spatially organized; for example, RNAPs of poliovirus form planar arrays/lattices with hundreds of molecules (6).

Transcription has also been shown to be spatially organized in bacteria, where early studies using conventional fluorescence microscopy in fixed cells showed that fluorescent derivatives of RNAP in *Escherichia coli* and *Bacillus subtilis* (7) form bright, diffraction-limited foci in rich medium, but not in minimal medium; these prokaryotic transcription foci have been likened to transcription factories (3,8). Subsequent studies using photo-activated localization microscopy (PALM), a super-resolution imaging method, provided further insight into RNAP spatial organization; using PALM on fixed cells under different growth conditions, it was shown that RNAPs form large clusters with ∼70 and >100 molecules in rich medium, and smaller clusters with ∼35 molecules in minimal medium (9). Single-molecule localization studies in live *E. coli* cells showed that RNAPs tend to co-localize with the nucleoid lobes, while being nearly absent from the ribosome-rich cell endcaps (10,11). Further live-cell work combining PALM and single-molecule tracking was able to distinguish between mobile RNAPs (i.e., RNAPs exploring the nucleoid for promoters) and immobile RNAPs, with the latter fraction including transcriptionally active RNAPs that localized primarily at the nucleoid periphery; this study also provided the first observation of RNAP clustering in living bacteria (12).

Surprisingly, subsequent localization-based work (13) suggested that RNAP clustering remained significant even when transcription was suppressed, and only decreased substantially when all transcription was inhibited by rifampicin, leading to the proposal that the underlying nucleoid (rather than high transcription activity) controls the organization of these RNAP clusters. Recently, it was suggested that RNAPs in bacteria form ‘biomolecular condensates’ (14) via liquid–liquid phase separation (LLPS), a phenomenon seen in many organisms (15–17), including bacteria (18–21); the condensates were shown to contain high-density RNAP clusters in fast-growth conditions, and were mediated by protein–protein interactions, offering LLPS as an alternative mechanism that drives RNAP clustering (22).

**Figure 1.**
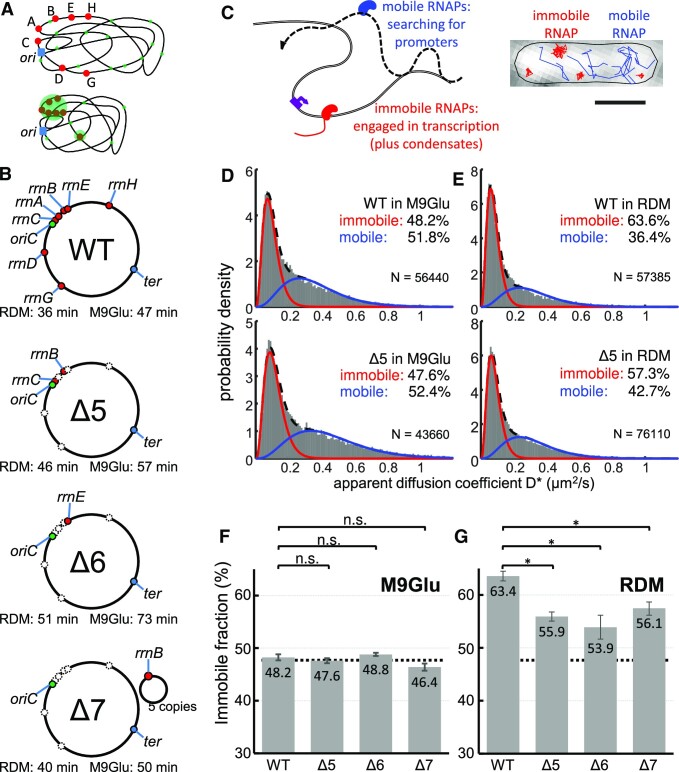
Measuring chromosome engagement of RNA polymerase at the single-molecule level in *E. coli* strains with deletions of most chromosomal *rrn* operons. (**A**) Models of RNAP distribution in cells based on observations of transcription foci and clustered RNAPs. Ribosomal operons are shown in red; RNAPs engaged in transcription are shown in green; the origin of replication is shown in blue. Top: transcription-engaged RNAPs in minimal medium are distributed throughout the nucleoid, with a bias towards the nucleoid periphery. Bottom: in rich medium, transcription is concentrated on *rrn* operons, most of which appear in close proximity, forming large RNAP clusters. (**B**) WT and *rrn* deletion strains used in this study; *rrn* loci are marked in the genomic map or in the supplemented plasmid. Doubling times of each strain in RDM and M9Glu medium are also shown. (**C**) Main mobility species of RNAP molecules and their characterization using single-molecule tracking PALM. Scale bar, 1 μm. (**D** and **E**) Histograms of the apparent diffusion coefficient (D*) fitted with two-gamma distributions for the WT and Δ5 in M9Glu (D) and RDM (E), along with the fractions of immobile and mobile RNAPs. N denotes the number of tracks per histogram. The immobile fraction includes RNAPs bound to the bacterial chromosome for several frames (12), while the mobile fraction corresponds to RNAPs interacting non-specifically and transiently with the entire chromosome (34). See the main text for details. (**F** and **G**) Fractions of immobile RNAP fractions for all strains in M9Glu (F) and RDM (G). Dashed lines, mean immobile RNAP fraction in M9Glu; error bars, SEM from three individual measurements. The differences in RDM are statistically significant (*P*< 0.05), whereas there are no significant differences in M9Glu.

A central point of debate in the spatial organization of transcription and the formation of transcription foci is the exact role of ribosomal rRNA operons (rRNA operons, *rrn*). rRNA transcription (which involves 16S, 23S and 5S rRNA) accounts for ∼85% of all active transcription in fast-growing cells (23); such high transcription levels are essential for sustaining rapid synthesis of the ∼55 000 ribosomes (10) needed per daughter per cell cycle during rapid growth (24). Notably, *rrn* transcription is much less prevalent in minimal medium. In the genomic map of *E. coli*, most of the seven *rrn* operons locate near the origin of replication (*oriC*), and all *rrn* operons orient in the same direction as DNA replication (Figure 1A); this chromosomal location leads to increased gene dosage for *rrn* genes.

Transcription foci and RNAP clustering have been linked to *rrn* operons even during the first RNAP distribution studies (1,9), which raised the possibility that transcription foci involve multiple (perhaps all) *rrn* operons operating in close proximity and in a growth-dependent manner (since such foci were absent in minimal medium), supporting a ‘bacterial nucleolus’ model (2,24). Consistent with that model, Gaal *et al.* measured the pairwise distance of *rrn* operons in single cells and found that six out of the seven *rrn* operons in *E. coli* are in close proximity in 3D space [Figure 1A; (25)]. Endesfelder *et al.* also linked RNAP clustering to *rrn* operons, and suggested that clusters with 35–70 molecules represent single *rrn* operons, and large clusters (>100 molecules; 50–300 nm in diameter) represent super-clustered multiple *rrn* operons (9). These indirect links were supported by Weng *et al.* (13), who directly showed that RNAP clusters were indeed co-localizing with sites of high *rrn* transcription in rich medium, while the formation of these clusters was independent of *rrn* transcription activity (13). The persistence of significant clustering despite the dramatic loss in *rrn* transcription was later attributed to LLPS (22).

Despite the progress in the understanding of spatial organization of transcription, there are still many open questions. What is the link between RNAP clusters and *rrn* operons, if any, in minimal medium? To what extent does LLPS contribute to RNAP clustering forming on *rrn* operons? What are the mechanisms that maintain the ability of cells to grow even when the number of chromosomal *rrn* operons is very small (1–2 copies)?

Here, we study the link between RNAP clusters and *rrn* operons by using single-molecule imaging and tracking (26,27) to obtain the RNAP spatial distribution and mobility in strains featuring deletions of most *rrn* operons (*Δrrn* strains; Figure 1B). We show that, remarkably, in strains with only one or two chromosomal *rrn* copies, bacterial cells maintain the same level (∼48%) of immobile RNAPs (which mainly reflects RNAPs engaged in transcription) during moderate growth rates; immobile RNAPs in *Δrrn* strains move close to cell endcaps, suggesting that RNAPs relocate to the remaining *rrn* operons, which have a pole-proximal location. During fast growth in rich medium, loss of most *rrn* operons leads to only a modest decrease of the immobile RNAP fraction, suggesting that RNAP redistributes to other *rrn* and non-*rrn* genes on the chromosome. RNAPs retained their clustering in the *Δrrn* strains, whereas co-localization analysis showed a good correlation between RNAP clusters and *rrn* operons. Our work expands our understanding of how RNAP is organized and allocated between transcription activities and how bacteria regulate their transcription to adapt to variations of their chromosomal content and growth environment.

## MATERIALS AND METHODS

### Bacterial strains

The *rpoC:PAmCherry* wild-type (WT) strain carrying PAmCherry fused to the β’ subunit under the control of its native promoter used was built as described previously (9). The *Δrrn* strains were obtained by P1 transduction of the *rpoC:PAmCherry* gene in the *Δrrn* strains. The deletion strains were acquired from CGSC (*E. coli* Genetic Stock Center at Yale): SQ88 as Δ5rrn with *rrn*B and *rrn*C remaining; SQ110 as Δ6rrn with *rrn*E remaining; and SQ2158 as Δ7rrn supplemented with plasmid-borne *rrn*B [pK4-16, based on pSC101, see also (28)].

Cell growth rate measurements were performed using OD_600_ on a microplate reader (FLUOStar, BMG Labtech). Three separate measurements were carried out with individual blank media. The absorbance of OD_600_ was measured every 5 min for 16 h to generate the growth curves.

### Cell preparation for imaging

Strains were streaked onto Luria–Bertani (LB) plates supplemented with required antibiotics for each strain. For the WT, we used 100 μg/ml ampicillin; for Δ5 and Δ6, we used 100 μg/ml ampicillin and 40 μg/ml spectinomycin, respectively; and for Δ7, we used 100 μg/ml ampicillin, 40 μg/ml spectinomycin and 50 μg/ml kanamycin. Single colonies were inoculated into LB and grown at 37°C and 220 rpm for a pre-culture of 2 h, then diluted 1/250 into M9Glu medium (1× M9 medium supplemented with CaCl_2_, MgSO_4_ and 0.2% glucose but without any additional vitamins or amino acids) or RDM (rich defined medium; Teknova) and grown at 37°C overnight. Overnight cultures were diluted into fresh medium and grown for > 2 h at 37°C until early exponential phase (OD 0.1–0.2 for M9Glu culture, or OD 0.2 for RDM culture). A 1.5 ml aliquot of cell culture was centrifuged down, concentrated to 30 μl and immobilized on 1% low-fluorescence agarose (BioRad) pads (supplemented with required M9Glu or RDM to keep media consistent). After immobilizing the cells on agarose pads with fresh medium, we monitored the RNAP localization using single-particle tracking by PALM at 22°C and measured the apparent diffusion coefficient (D*) of RNAPs (see also Figure 1C, D).

For fixed-cell co-localization experiments combining PALM with fluorescence *in situ* hybridization (FISH), 1.5 ml of culture of the WT or Δ*rrn* strains carrying rpoC:PAmCherry were spun down and then resuspended into 1 ml of phosphate-buffered saline (PBS). A 1 ml aliquot of 4% paraformaldehyde (PFA) was mixed 1:1 with the bacterial culture and incubated for 40 min with mild shaking on a nutator mixer at room temperature. After three washes with PBS, we added 500 μl of absolute ethanol to permeabilize the cells, and washed them twice with PBS. We then immobilized 20 μl of cells on chitosan (29) housed in a self-adhesive gasket. For the FISH studies, the pre-rRNA probes (5 μM) carrying the sequence [Atto488]TGCCCACACAGATTGTCTGATAAATTGTT AAA-GAGCAGTGCCGCTTCGCT (13) were incubated with the permeabilized cells and incubated for 5 min at room temperature, then washed three times with PBS. Cells were then imaged as discussed below.

### PCR-based measurement of the *ori:ter* ratio

The *ori:ter* ratio, which provides a measure of the rate of DNA replication initiation, was measured from genomic DNA extracted from strains with a different number of *rrn* operons and in different media. The procedure was performed as described (30–32) with minor modifications. Briefly, cells were grown overnight in either RDM or M9Glu at 37°C. The following morning, cells were diluted 1:100 in 30 ml of the corresponding starting medium until OD_600_ ∼0.2–0.25; 20 ml of the cultures were then spun down, and the pellets were frozen at –80°C. The pellets were treated with 5 mg/ml lysozyme and RNase, and the DNA was extracted using the phenol–chloroform method. The purified DNA was digested by EcoRI, and 100 ng was used in the polymerase chain reaction (PCR). The origin and terminus regions were amplified utilizing oligos in *gidA* and *dcp* genes, respectively, using sequences found in (30,31). The PCR was set up with Sso Advanced Universal SYBR Green Supermix (Biorad) in 20 μl reactions and carried out on an ABI 7500 Fast Real-Time PCR system (Thermo Scientific) under the cycling conditions in (31) with the exception that the initial denaturation temperature was kept at 98°C for 120 s. The *ori:ter* ratio was calculated using the comparative cycle threshold (Ct) analysis method utilizing the 2^−ΔCt^ approach (33). The data represent the mean and standard error of the mean (SEM) of three technical repeats.

### Single-molecule imaging of living cells

A custom-built single-molecule tracking photo-activated localization microscope (12) was used for the imaging of single RNAP–PAmCherry molecules and the detection of diffraction-limited *rrn* foci. Cells mounted on 1% agarose pads were imaged under bright-field illumination to perform cell segmentation. Prior to PALM imaging, pre-activated PAmCherry molecules were photobleached under continuous 561-nm excitation. Sparse photoactivation of the remaining population of PAmCherry was performed by continuous exposure to low intensity 405-nm excitation, such that the dataset consists of well separated single molecules. Under simultaneous excitation with a 561-nm laser, these photoactivated molecules fluoresce until permanently photobleached. Imaging was performed at a frame rate of 15 ms/frame for at least 30 000 frames, until the entire pool of RNAP–PAmCherry molecules had been imaged.

### Two-colour co-localization assay

For FISH imaging of *rrn* foci with fixed cells, a 488-nm laser was used for 20 frames at 500-ms exposures. Brightfield imaging and pre-bleaching with a 561-nm laser were performed as for live cells. Excitation of RNAP–PAmCherry molecules was performed using a 561-nm laser for 90 000 frames at 15 ms/frame to capture the entire pool of RNAP molecules.

### Image processing and data analysis

Live-cell data were processed following published procedures using custom-written MATLAB software (12) for localizing single RNAP–PAmCherry. Briefly, individual frames of the PALM video were processed to obtain the approximate positions of molecules. The precise location of each molecule was further refined by fitting a 2D elliptical Gaussian function to each of these candidate positions. The molecular trajectories were obtained by linking these localizations in successive frames. In rare cases for which multiple localizations are present simultaneously within this search radius, localizations were linked to their closest counterpart in the following frame. If a localization is absent from a single frame of the trajectory (e.g. as a consequence of fluorophore blinking), the localizations on either side of the empty frame were connected.

A histogram of the apparent diffusion coefficient, D*, was compiled for each dataset by computing the mean squared displacement (MSD) of individual trajectories using at least four single-step distances. Histograms of the apparent diffusion coefficient were fitted with two-gamma distributions, with the value for the immobile species fixed at a value measured using an experimental control [0.08–0.10 μm^2^/s, based on DNA polymerase I measurements, as in (12)], and the value of the mobile species left unconstrained. This fitting routine allowed us to accommodate conditions where the presence of larger amounts of chromosomal DNA in the cell (e.g. as we move from M9Glu to RDM) leads to a D* decrease due to more pronounced RNAP non-specific DNA binding [which effectively decreases RNAP mobility; (34)]. Comparison of different strains in defined growth medium was done by collecting data in triplicate. Fixed cell data were processed using rapidSTORM as previously (9,35) to localize single RNAP–PAmCherry molecules while removing any repeated localizations in flanking frames.

### Clustering analysis of RNAP localizations

Clustering analysis of RNAP molecules localized by rapidSTORM was done using a MATLAB implementation of the DBSCAN algorithm [see (9,36) and Yarpiz page: Mostapha Kalami Heris, DBSCAN Clustering in MATLAB (URL: https://yarpiz.com/255/ypml110-dbscan-clustering), Yarpiz, 2015]. Clusters of molecules were identified by constructing a coordinate list of the first localization from each trajectory. The DBSCAN algorithm operates on this coordinate list using two parameters, ϵ and MinPts, to categorize the localizations into three groups. Any localization in a region containing at least MinPts localizations, including itself, within the distance ϵ are classified as ‘core’ localizations. Localizations that do not meet these criteria, but fall within the distance ϵ of a core localization, are classified as ‘directly reachable’, while the remaining localizations are classified as ‘noise’. Using these classified localizations, a cluster of molecules is then defined as any group of connected core and directly reachable localizations. From a Monte-Carlo simulation of localizations of RNAPs in M9Glu medium and based on previous measurements in fixed cells (9), we determined the appropriate parameters for reliable clustering to be ϵ = 20 nm and MinPts = 4.

While the DBSCAN algorithm identifies individual clusters present in a cell, we also require a global picture of clustering for each dataset that is also ideally independent of input parameters. For a parameter-free quantitative description of clustering, we therefore use the pair correlation function, which describes how the density of molecules varies as a function of distance from a reference molecule. In the case of RNAP localizations, the pair correlation function is evaluated by computing the Euclidean distances between all pairs of molecules in a single cell, and binning the results into a histogram of evenly spaced intervals. The segmented cell boundary is then used to generate a uniform (non-clustered) distribution of the same number of molecules throughout the cell volume. The distribution obtained from the experimental result is then normalized by this simulated distribution, to produce the pair correlation function. This process of normalization eliminates artefacts from the confining geometry of the cell. To avoid projection effects, the simulated molecules were distributed in a 3D volume generated by rotating the segmented cell boundary around its long axis, and then projected into 2D by removing one of these dimensions. In the resulting pair correlation function, g(r), molecules distributed evenly throughout the cell result in a flat distribution with g(r) = 1 for all values of r, whereas a population of molecules exhibiting clustering results in g(r) > 1 at short distances, and g(r) < 1 at long distances.

### Pair correlation of RNAP clusters with *rrn* foci

Analysis of *rrn* foci in fixed cells was performed by localizing both the *rrn* foci and RNAP–PAmCherry molecules by applying a bandpass filter and intensity threshold to identify molecules, followed by free elliptical Gaussian fitting to obtain high-precision localizations. The pair correlation g(r) function was computed for all RNAP–*rrn* distances within the cell. According to (37), the nucleoid area covers ∼56% of the overall cell area in M9Glu medium; therefore, we shrink both the cell length and width to ∼75% to estimate the nucleoid area. A second uniform distribution was then generated and normalized using the same distribution as the experimental data to provide a visual guide for completely uncorrelated data, shown as the dashed line in the plots of the pair correlation function. Finally, the fraction of molecules found within 200 nm of *rrn* foci was calculated for both the experimental and uniform data.

### Heatmap plotting

Cell boundaries were determined from brightfield images using the software microbeTracker to obtain the spatial location of individual molecules relative to the major and minor cell axes. These spatial locations were binned into a 2D histogram normalized by the cell length and width, to produce a heatmap which visualizes the spatial density of molecules. Heatmaps were produced for cells containing single and double nucleoids by applying a threshold for cell length (Supplementary Figure S2). Heatmaps were further subcategorized into mobile and immobile molecules by a threshold for diffusion coefficient. Finally, a heatmap illustrating the difference between the immobile and mobile heatmaps was generated by subtracting each element of the mobile heatmap from the immobile heatmap. Heatmaps were additionally projected along their long and short axes to illustrate the RNAP distribution throughout the cell volume.

### Simulations

The 3D simulations of RNAP molecule locations were performed using Monte-Carlo methods for the WT, Δ5 and Δ6 strains. RNAP molecules were categorized into four populations; mobile; bound to *rrn* operons at the nucleoid periphery; bound in small clusters throughout the nucleoid; and ‘noise’ found throughout the cell volume. A total of 1800, 1800 and 1200 molecules were distributed between these categories in the WT, Δ5 and Δ6, respectively, consistent with experimental results in Supplementary Figure S9. A split of 48% immobile, 52% mobile molecules was simulated based on experimental data presented in Figure 1.

A probability density function for non-*rrn*-associated small RNAP clusters was obtained by fitting cluster size distributions obtained experimentally to an exponential function of the form y = ae^−bx^. The simulated population of non-*rrn* small cluster sizes are then generated by sampling from the inverse transform of this exponential probability density function, X = –(1/b)ln[1 – (bu/a)], where u[0:1] is a set of uniformly distributed random numbers. The centres of non-*rrn*-associated RNAP clusters were distributed uniformly throughout the nucleoid volume, around which RNAP molecules were distributed isotropically with a Gaussian radial density profile. Any molecules generated outside of the modelled cell volume were regenerated until a complete distribution was obtained.

The mobile population distributed uniformly within the nucleoid was generated via rejection sampling within a prolate spheroid volume positioned with a 150 nm separation between the nucleoid and cell poles. The same code was used to position the centres of small (non-*rrn*) clusters. A fraction of the mobile population was diverted to a population of localization ‘noise’ that was generated uniformly across the entire cell volume via rejection sampling.

*rrn* operons were positioned along the pole-proximal periphery of each nucleoid. Simulations were performed with seven operons/nucleoid in the WT, two in Δ5 and one in Δ6. The location of each cluster centre along the long axis was weighted by the genomic distance of each *rrn* operon relative to *oriC*, defining a ring of possible locations around the nucleoid periphery. Candidate locations around this ring were proposed until a position was obtained with a minimum separation of at least 70 nm from other *rrn* operons. RNAP molecules were then distributed around each of these *rrn* cluster centres isotropically as described above for small non-*rrn* clusters. The proportion of immobile molecules associated with *rrn* operons was simulated across the range of 30–80%, with the value of 60% most closely matching the experimental data in Figure 4. These 3D simulations were then projected into 2D, and analysed by computing the pair correlation function g(r) for each cell. The process was then automated for 2000 cells to obtain the mean g(r) for the distribution.

### Estimation of the copy number of *rrn* operons and rRNAPs for a given growth rate

The total number of RNAPs engaged with the *rrn* operons (N_r_) for each strain was estimated by interpolation of the N_r_ values from (38) [which were calculated using the expression N_r_ = r_r_/c_r_, where r_r_ and c_r_ are the overall rate of rRNA synthesis and the rRNA elongation speed (85 nt/s) as measured by Bremer and Dennis (39)] by fitting to a single exponential. The expected number of *rrn* operons in all strains for a given growth rate was obtained as in Bremer and Dennis [equation 9 in table 5 of (39)], by considering the growth rate of each strain and the location of each *rrn* on the map of the *E. coli* chromosome.

## RESULTS

### RNAPs remain heavily engaged with the chromosome despite deletion of most *rrn* operons

To clarify the relationship between RNAP clusters and *rrn* operons, we compared a well characterized *E. coli* strain carrying all seven chromosomal *rrn* operons (‘wild type’, WT) with strains carrying a drastically reduced number of *rrn* operons; these *rrn* deletion strains (*Δrrn*) were originally developed to study the link between *rrn* operon multiplicity and ribosome function (40,41).

Specifically, we studied a strain in which five out of seven operons were deleted, leaving only *rrnB* and *rrnC* on the chromosome (Δ5, Figure 1B); a strain in which six out of seven operons were deleted, leaving only *rrnE* on the chromosome (Δ6, Figure 1B); and a strain in which all seven chromosomal *rrn* operons were deleted, and instead supplemented by a low copy-number plasmid (∼5 copies per chromosome) containing a single *rrnB* operon (Δ7, Figure 1B). To enable tracking of single RNAP molecules in cells, all strains contained a fully functional C-terminal fusion of the β’ subunit of RNAP with a photoactivatable mCherry (PAmCherry (9,12); see also the Materials and Methods).

To check the fitness of *Δrrn* strains relative to the WT, we monitored their growth in different media (Figure 1B; Supplementary Figure S6); in general, the growth rates in the deletion strains correlated to the number of remaining copies of *rrn* operons, with Δ6 being the slowest growing. The WT strain displayed a 47 min doubling time at 37°C in minimal M9 medium supplemented with 0.2% glucose (M9Glu; see the Materials and Methods); in comparison, Δ7 grew marginally more slowly (50 min), Δ5 grew significantly more slowly (57 min) and Δ6 grew substantially more slowly (73 min, Table 1). The growth rates of *Δrrn* strains in rich medium followed a similar pattern; in RDM, the WT was the fastest growing (36 min), followed by Δ7, Δ5 and Δ6 (40, 46 and 51 min, respectively; for LB, see Supplementary Figure S6). In general, reduction in the number of *rrn* copies led to a small to moderate decrease in the growth rate, presumably by affecting the rate at which different strains produce ribosomes (24,42).

**Table 1.** Estimated occupancy of *rrn* operons in WT and *Δrrn* strains as a function of growth rate

Strain	Medium	Doubling time (min)	N_rRNAP_ per cell^a^	*oriC* copies per cell	*rrn* copies per cell	N_rRNAP_ per *rrn*	RNAP occupancy of each *rrn*^b^
WT	M9Glu	47	397	2.9	17.5	23	32%
Δ5	M9Glu	57	238	2.5	4.8	50	69%
Δ6	M9Glu	73	122	2.2	2.0	61	85%
WT	RDM	36	805	3.7	22.0	37	51%
Δ5	RDM	46	420	2.9	5.6	75	104%
Δ6	RDM	51	320	2.7	2.5	128	178%

For the methods used to obtain the estimates for growth at 37°C, see the Materials and Methods.

^a^Number of RNAP molecules transcribing *rrn* operons in the different strains.

^b^100% occupancy is defined as the RNAP occupancy at the maximum growth rate (72 rRNAPs/*rrn*). Note that this occupancy is substantially lower than the maximal physical occupancy (see also the Discussion).

To follow the RNAP mobility in live cells, we performed single-particle tracking of RNAP molecules using PALM on surface-immobilized cells, as described [(12); see also the Materials and Methods]. The single-molecule tracks allowed us to calculate apparent diffusion coefficients (D*) for hundreds of RNAPs per cell (Figure 1C–E; see also the Materials and Methods) and construct D* histograms (for the number of RNAP localizations in our growth media, see Supplementary Figures S7 and S8). The D* distribution for RNAP in WT cells grown in M9Glu was described well by two RNAP fractions with different mobility: a species (48.2% of all tracks) that remains immobile (D* of ∼0.09 μm^2^/s) and a mobile species (51.8% of all tracks) with D* of ∼0.36 μm^2^/s (Figure 1D, top; Supplementary Figure S1A, top). The immobile fraction includes RNAPs bound to the bacterial chromosome for several frames (12), which in turn correspond mainly to RNAPs bound to promoters and transcribed genes; the immobile species includes any RNAPs found in condensates, since they have been suggested to possess very low mobility (22). On the other hand, the mobile fraction corresponds to RNAPs interacting non-specifically and transiently with the entire chromosome during their promoter search (34).

To assess the effect of *rrn* operon loss on RNAP mobility in M9Glu, we compared the D* distribution of the WT with those of *Δrrn* strains (Figure 1D, F; Supplementary Figure S1C). In Δ5, and despite the deletion of five out of seven chromosomal *rrn* operons, the D* distribution and the immobile RNAP fraction were essentially identical to those in the WT (47.6%; Figure 1D, bottom; Figure 1F). The lack of any significant mobility difference compared with the WT was also observed for both Δ6 and Δ7 strains (48.8% and 46.4%, respectively; Figure 1F; Supplementary Figure S1B, C, left). These results establish that, despite the loss of most *rrn* operons, *Δrrn* strains show the same level of immobile RNAP as the WT.

To assess the effect of *rrn* operon loss on RNAP mobility in RDM, where the *rrn* operons should be much more heavily occupied by RNAPs than mRNA-coding genes [and where any RNAP condensates should be more visible, potentially accounting for ∼30% of the immobile fraction; (22)], we performed similar RNAP mobility comparisons between WT and *Δrrn* strains in RDM. As we observed before (12), the immobile RNAP fraction in the WT was ∼63% (Figure 1E, top; Figure 1G), while Δ5 showed an ∼6% decrease in the immobile fraction (57.3%; Figure 1E, bottom; Figure 1G; Supplementary Figure S1C, right); the results for Δ6 and Δ7 showed a similar decrease in immobile RNAPs (53.9% and 56.1%; Figure 1G; Supplementary Figure S1B, C, right). In general, for all *Δrrn* strains grown in rich medium, the immobile RNAP fraction stays surprisingly at the same high level (54–56% on average).

### DNA-bound RNAPs relocate to pole-proximal positions in *Δrrn* strains in M9Glu

**Figure 2.**
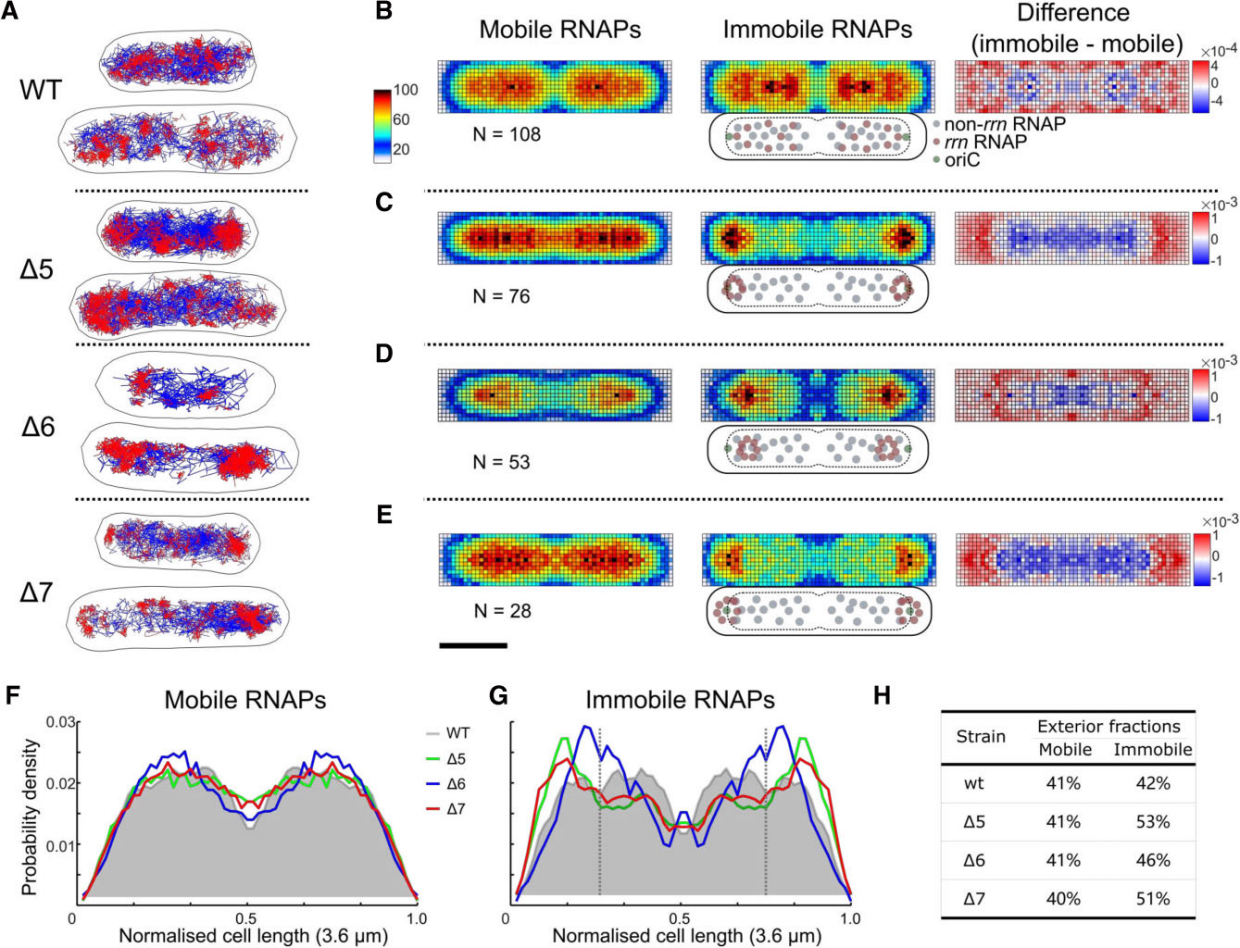
Immobile RNAPs in the *Δrrn* strains redistribute to polar-proximal regions in M9Glu medium. (**A**) Tracks of immobile (red) and mobile (blue) RNAP molecules for representative cell examples. The D* threshold for the track colour-coding was 0.16 μm^2^/s. Scale bar (shown in D), 1 μm. (**B**–**E**) Left and middle: heatmaps from multiple cells within 3.2–4.0 μm range of cell lengths for both mobile and immobile RNAPs; the colour scheme denotes the number of RNAPs per pixel (blue for a low number, and red for a high number; see also heat map legend). Right, difference map calculated by subtraction of mobile RNAPs from immobile RNAPs; the colour scheme denotes the over- or under-representation of immobile RNAPs per pixel (red for over-representation, and blue for under-representation; see also heat map legend). Cartoons below the immobile RNAP heatmaps display the relative positions of *rrn*-related and non-*rrn*-related RNAPs relative to the cell membrane, nucleoid and the *ori* region. Scale bar, 1 μm. (**F** and **G**) Projections of mobile (F) and immobile (G) RNAP localizations along the long axis of the heatmaps. The projection from the WT is shaded grey. Dashed vertical lines indicate the 25% position along the long axis. Notably, Δ6 shows a less pole-proximal location, probably reflecting that fact that the position of the remaining *rrn* operon in Δ6 (*rrnΕ*, 306 kb from *oriC*) is further away from *oriC* compared with the two *rrn* operons remaining in Δ5 (*rrnC* and *rrnB*, 42 kb and 265 kb from *oriC*, respectively); considering the ori–ter (longitudinal) organization of the bacterial chromosome during slow growth (with *oriC* being close to the cell pole and the terminus near the mid-cell), any RNAP relocation to *rrnE* in Δ6 should provide less pole-proximal positions versus Δ5 and Δ7 (with the latter containing *rrn* operons on plasmids, which tend to have a polar localization). (**H**) The fractions of RNAPs localized in the exterior 25% region along the long axis (i.e. from 0 to 0.25 normalized cell length in F).

To examine whether the deletion of most or all chromosomal *rrn* operons leads to any RNAP relocation within cells, and to gain insight regarding any redistribution between cellular RNAP pools, we examined the spatial distributions of mobile and immobile RNAPs in *Δrrn* strains via sorting single-molecule tracks using a D* threshold [Figure 2A, B; see also (12) and the Materials and Methods). To capture the average behaviour for cells of similar size, we pooled the normalized positions of RNAPs from individual cells within different size ranges, and generated spatial heatmaps for both mobile and immobile fractions (see the Materials and Methods).

As we observed previously (12), the RNAP spatial distribution in WT cells with two nucleoids in M9Glu showed that mobile RNAPs localize throughout the nucleoid, essentially highlighting the nucleoid location (Figure 2B, left), whereas immobile RNAPs tended to localize at the nucleoid periphery [Figure 2B, middle; see also (12)]; this redistribution towards the periphery for immobile molecules can be seen more clearly in the normalized difference heatmap between the two mobility fractions (Figure 2B, right). Similar results were obtained for shorter cells, which carry only a single nucleoid (Supplementary Figure S2).

We then examined strain Δ5 to see how the deletion of five *rrn* operons affects the RNAP spatial distribution. The mobile RNAPs in Δ5 had a spatial distribution nearly identical to that of the WT, i.e. spanning the entire nucleoid (Figure 2C, left), reflecting the transient, non-specific interactions of this target-searching RNAP fraction with the nucleoid (34). In contrast, the spatial distribution of immobile RNAPs in Δ5 is substantially different from that of the WT, with immobile RNAPs becoming much more concentrated at the pole-proximal edges of the nucleoid (Figure 2C, middle; see also the difference heatmap, Figure 2C, right). For a clearer view of this RNAP relocation, we projected the heatmaps along the cell length (Figure 2F, G). While the projection for mobile RNAPs is similar, the projection of immobile RNAPs shifts from a fairly flat distribution centred at ∼30% of cell length for the WT, to a distribution with a peak at ∼15% of cell length for Δ5 (green line, Figure 2G); this shift is also reflected by the large increase in the RNAP fraction localized in the exterior 25% region along the long axis (53% in Δ5 versus 42% in the WT; Figure 2H); this increase corresponds to the relocation of ∼10% of all immobile RNAPs.

A profile similar to that of Δ5 was observed for Δ7, which features only plasmid-borne *rrn* operons: the mobile RNAPs cover the entire nucleoid (Figure 2E, left), while ∼10% of the immobile RNAPs relocate to pole-proximal regions (Figure 2E, middle and right; Figure 2H), shifting the peak in the projection of immobile RNAPs to ∼15% of cell length (Figure 2G, red line). The Δ6 strain (with a single chromosomal *rrn* operon) also behaved similarly to Δ5 and Δ7 for both mobile and immobile RNAPs (Figure 2D, F, G), with the main differences being the position of the new peak of the immobile fraction, which appears at ∼20% of cell length (Figure 2G, blue line). Similar results were obtained for shorter cells (Supplementary Figure S3).

To explain our spatial distributions of immobile RNAPs, we need to consider that they contain several RNAP pools: RNAPs transcribing *rrn* operons (rRNAPs), RNAPs transcribing mRNAs (mRNAPs) and any condensate-associated RNAPs (cRNAPs). Removal of several *rrn* operons from the chromosome essentially releases many rRNAPs; since the immobile fraction for all three *Δrrn* strains does not change relative to the WT, the released rRNAPs must join one or more of the three main pools of immobile RNAPs. Since the *Δrrn* strains do not have a substantial growth defect, it is likely that to maintain sufficient rRNA synthesis to support ribosome biogenesis, many (perhaps all) of the released rRNAPs are captured by the remaining *rrn* operons (see also the Discussion). Our results also show that a large fraction of immobile RNAPs in the *Δrrn* strains engage with the entire nucleoid (Figure 2C–E, middle); we attribute this fraction mainly to non-*rrn*-associated immobile RNAPs.

### Immobile RNAPs spread throughout the nucleoid in rich medium

**Figure 3.**
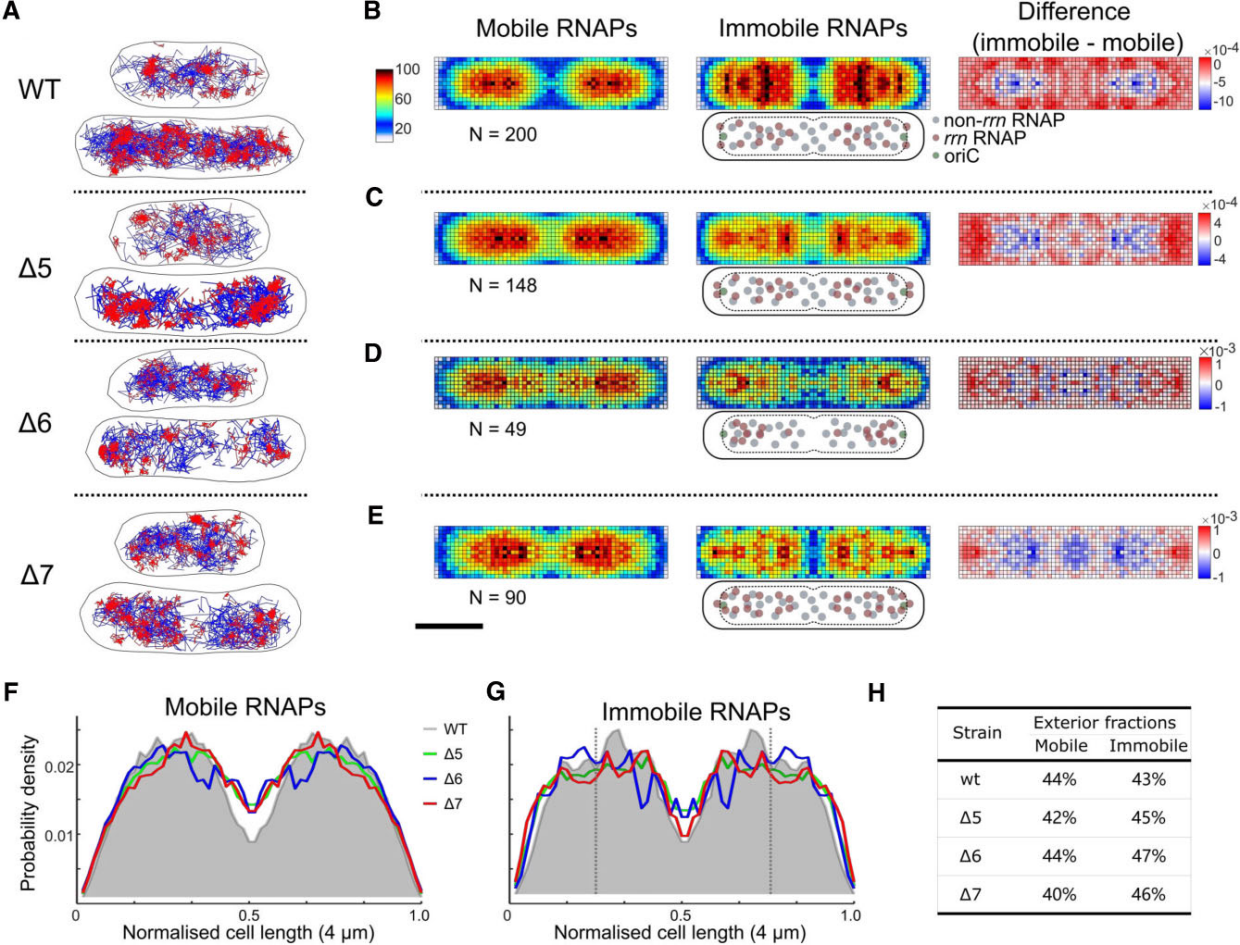
RNAP spatial distribution in the WT and *Δrrn* strains in rich medium (RDM). (**A**) Tracks of immobile (red) and mobile (blue) RNAP molecules for representative cell examples. The D* threshold for the track colour coding was 0.16 μm^2^/s. Scale bar (shown in D), 1 μm. (**B–E**) Left and middle: heatmaps from multiple cells within a 3.5–4.5 μm range of cell lengths for both mobile and immobile RNAPs; colour scheme as in Figure 2B. Right: difference map calculated by subtraction of mobile RNAPs from immobile RNAPs; colour scheme as in Figure 2B. Cartoons below the immobile RNAP heatmaps display the positions of *rrn*-related and non-*rrn*-related RNAPs relative to the cell membrane, nucleoid and the *ori* region. Scale bar, 1 μm. (**F** and **G**) Projections of mobile (F) and immobile (G) RNAP localizations along the long axis of the heatmaps. The projection from the WT is shaded grey. Dashed vertical lines indicate the 25% position along the long axis. (**H**) The fractions of RNAPs localized in the exterior 25% region along the long axis (i.e. from 0 to 0.25 normalized cell length in F).

Since growth conditions dramatically influence the RNAP spatial distribution (1), we examined the spatial distribution in *Δrrn* strains growing exponentially in RDM (Figure 3), a condition wherein cells need to accumulate high numbers of ribosomes [up to 70 000; see also (41)] and thus require high *rrn* expression (43,44). In RDM, WT cells divided every ∼36 min, a growth rate that corresponds on average to ∼2.7 chromosomes/cell, 3.7 replication origins per cell and a high copy of *rrn* genes [∼22 *rrn*/cell; (38,39); Table 1]. The RNAP distribution in the WT strain in RDM showed that, as in M9Glu, mobile RNAPs explore the entire nucleoid, whereas many immobile RNAPs appear in clusters distributed throughout the nucleoid (see the next section), with some enrichment at the nucleoid periphery (Figure 3A, B; Supplementary Figure S4); this enrichment, especially visible along the short cell axis, is clear in the difference map between immobile and mobile populations, for both long and short cells (Figure 3B, right; Supplementary Figure S4).

In contrast to its profile in M9Glu, the Δ5 strain shows a profile similar to the WT for both mobile and immobile RNAPs, i.e. both populations are evenly distributed along the long axis of cells, and much of the immobile population seen in single cells appears clustered in a few foci (Figure 3A, second row). Intriguingly, and in contrast to the profile in M9Glu, no apparent relocation of immobile RNAPs to the poles is observed (Figure 3C; Supplementary Figure S4), with the RNAP fraction in the pole-proximal region along the long axis being essentially identical for Δ5 and the WT, for both mobile and immobile RNAPs (Figure 3F, G). Broadly similar profiles and observations were seen in Δ7 (Figure 3A, bottom; Figure 3E) and Δ6 (Figure 3A, D), as well as in populations of short cells (Supplementary Figure S5). Notably, long cells feature a more polar and ‘nucleoid-excluded’ localization of immobile RNAPs in Δ7 relative to the mobile population (Figure 3E), probably reflecting the localization of most plasmids.

To explain the spatial distributions of immobile RNAPs, we consider that, during fast growth conditions in rich medium, cells contain multiple copies of the chromosome (9,45) and multiple sets of *rrn* operons, with the *ori*-proximal location of *rrn* genes further increasing the number of *rrn* copies (46); for example, for Δ5, we expect the group of long cells to have ∼3 chromosomes and ∼8 *rrn* copies on average (39). As in M9Glu, removal of several *rrn* operons from the chromosome releases many rRNAPs; since the bound fraction for all three *Δrrn* strains in RDM decreases only by ∼6% relative to the WT, the released rRNAPs must join one or more of the three main pools (rRNAPs, mRNAPs and cRNAPs). We reason that, to maintain sufficient rRNA synthesis to support ribosome biogenesis (albeit at reduced growth rates), most of the released rRNAPs in *Δrrn* strains are re-captured by the remaining *rrn* operons (see also the Discussion), whereas the remainder join the mRNAP pool.

Our interpretation above is consistent with the location of RNAP clusters in single cells (e.g. Δ5 cells in Figure 3A), which roughly map to the expected location of the four replication origins for cells of this size and growth rate; notably, the remaining *rrn* operons in Δ5 are proximal to *ori*. However, since the group of long cells covers a range of lengths (3.5–4.5 μm), and since the location of *rrn* operons varies for cells of different length, the average picture for the group of long cells is blurred and features fairly continuous distributions that do not reflect the localized nature of the clusters seen in single cells. On the other hand, Δ7 shows a clear profile of nucleoid exclusion for a large fraction of immobile RNAPs, suggesting that these represent RNAPs transcribing *rrn* genes on nucleoid-excluded plasmids. These *rrn*-centred RNAP pools are in addition to any pools of cRNAPs, although it is unclear whether cRNAP pools nucleate on the *rrn*-centred RNAP pools, or exist in isolation.

### RNAP clustering increases upon loss of most chromosomal *rrn* operons

**Figure 4.**
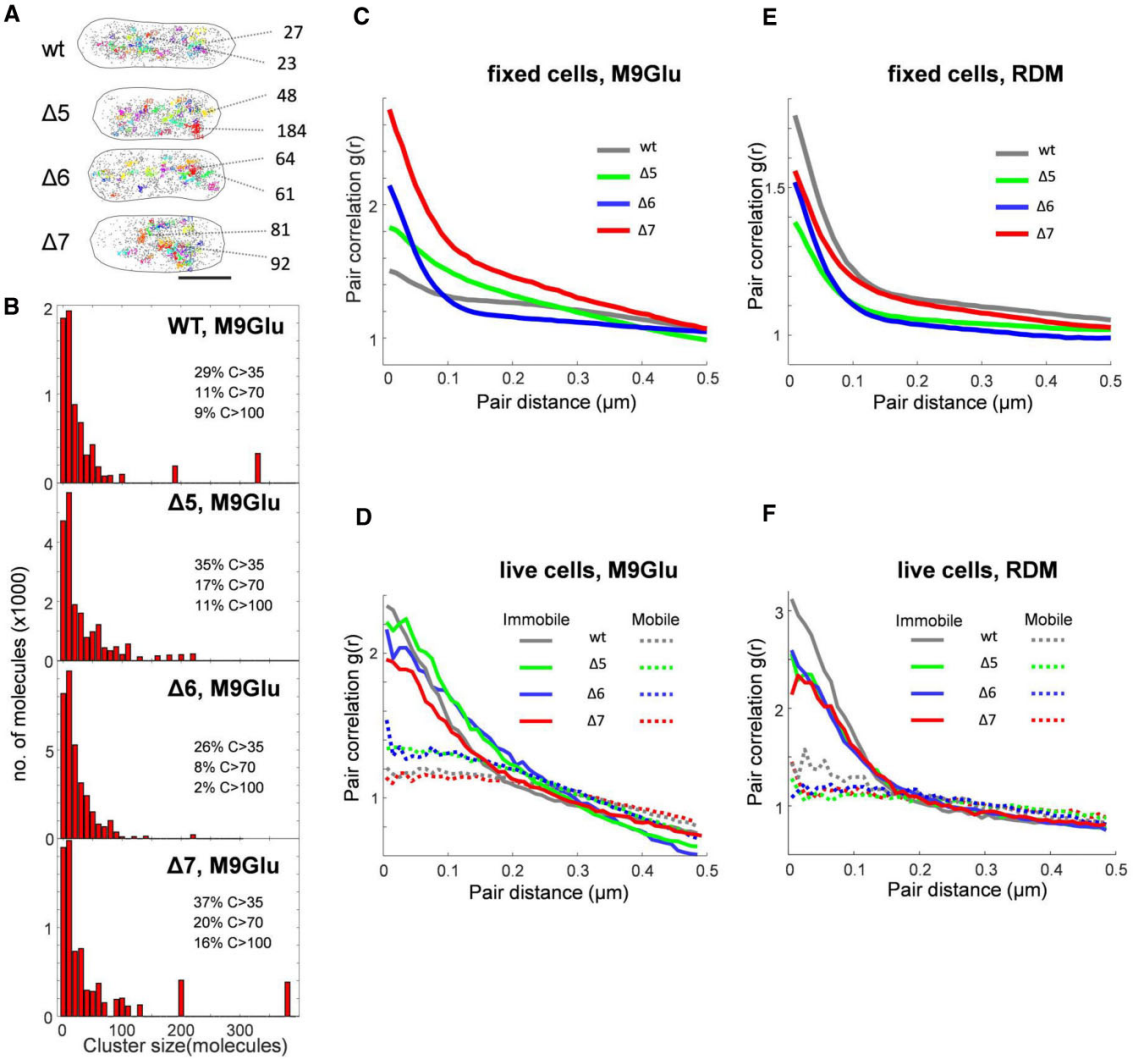
RNAP clustering and pair correlation analysis of RNAP localizations in M9Glu and rich media. (**A**) Representative examples of RNAP clusters in single fixed cells for the WT and two *Δrrn* strains. RNAP clusters are displayed in different colours; non-clustered localizations are displayed as isolated grey points. Scale bar, 1 μm. Two clusters from each cell along with the cluster size (number of molecules) are shown as examples. (**B**) Histograms of cluster size of RNAPs in fixed cells in M9Glu medium. The fractions of large clusters of different size are listed alongside. (C–F) Pair correlation analysis of RNAP localizations in fixed and live cells of WT and *Δrrn* strains in M9Glu and RDM. (**C**) Analysis in fixed cells of all strains in M9Glu. (**D**) Analysis of immobile and mobile RNAP tracks in live cells for all strains in M9Glu. (**E**) Analysis of RNAP localizations in fixed cells for all strains in RDM. (**F**) Analysis of immobile and mobile RNAP tracks in live cells for all strains in RDM.

The RNAP spatial distribution in *Δrrn* established that RNAPs in M9Glu relocate in pole-proximal regions, raising the possibility that relocation forms new clusters or enlarges smaller ones. To assess the level of RNAP clustering, we performed clustering analysis using the DBSCAN algorithm [(36) and see the Materials and Methods]. Analysis of the WT grown in M9Glu and subsequently fixed (Figure 4A, top) showed that 29, 11 and 9% of clustered RNAPs were found in clusters with >35, >70 and >100 molecules, respectively (C > 35, C > 70 and C > 100 species, Figure 4B, top) (9). For the Δ5 strain in M9Glu medium, which shows an RNAP copy number per cell similar to that of the WT (1767 versus 1844), we detected a significant increase in all three species of large clusters (Figure 4B), and notably for the C > 70 population (17% for Δ5 versus 11% for the WT), suggesting that RNAP redistribution increases the abundance of large clusters. Similar results were observed for Δ7, where despite a lower measured RNAP copy number for Δ7 (1285 versus 1844 for the WT), the C > 70 population increases to 20% (cf. 11% for the WT; Figure 4B, bottom). Interestingly, there is a small reduction in large clusters in Δ6, in part due to a decrease in the copy number of RNAPs (1140; Supplementary Figure S9; see also Supplementary Figure S10 for the same number for fixed cells grown in rich medium). These results are consistent with the deletion of most *rrn* increasing the degree of clustered RNAP due to relocation of RNAPs to the remaining *rrn* copies, all found in pole-proximal regions.

To gain another perspective to RNAP clustering, we performed pair correlation analysis of the RNAP localizations (12), wherein the distances between all pairs of individual molecules are analysed and compared with a random distribution; a pair correlation g(r) value of >>1 for a range of intermolecular distances indicates significant clustering, whereas g(r) ∼1 indicates a non-clustered distribution. Notably, the pair correlation analysis is unaffected by differences in RNAP copy numbers per strain, and requires no optimization in analysis parameters. We first examined RNAPs in fixed cells grown in M9Glu, and observed that RNAPs in the WT show only slight clustering at distances within ∼100 nm, whereas all three *Δrrn* strains showed much higher clustering within the ∼100 nm range (Figure 4C), with Δ7 being the most clustered strain. Pair correlation analysis on simulated data for the two strains retaining chromosomal *rrn* copies (Δ5 and Δ6; see the Materials and Methods) also showed that the WT is expected to maintain the lowest level of RNAP clustering relative to Δ5 and Δ6 (Supplementary Figures S11 and S12), consistent with our experimental results.

We also performed pair correlation analysis in live cells in M9Glu. These experiments are complicated by any 3D motions of clustered RNAPs during the ∼8 min of imaging; such motions will reduce the pair correlation and spread it out to longer length scales; however, any persistent clustering should still be visible. Since we can separate the mobile and immobile RNAP species, we performed pair correlation analysis of the two species separately. Since we do not anticipate mobile RNAPs to be clustered (apart from exploring the entire nucleoid; as such, they do not fill the entire cell), this analysis should offer clearer views of the clustering of immobile RNAPs. Indeed, mobile RNAPs for all strains do not cluster (Figure 4D, dotted lines); in contrast, the immobile RNAPs of both WT and *Δrrn* strains (Figure 4D, solid lines) appear much more clustered than mobile RNAPs. Further, all strains show a similar level of clustering for immobile RNAPs. Taken together, our results indicate that in M9Glu, RNAPs become more clustered, consistent with the remaining *rrn* operons in *Δrrn* strains accommodating relocated RNAPs to compensate the loss of many chromosomal *rrn* operons.

In rich medium, RNAPs in fixed cells of the WT are more clustered than in M9Glu, whereas, in contrast, RNAPs in all *Δrrn* strains show reduced clustering relative to their levels in M9Glu, and relative to the WT in RDM (Figure 4E; see Supplementary Figure S10 for the number of RNAPs per cell in RDM). Pair correlation analysis on the immobile RNAP molecules in live cells shows similar differences between the WT and the *Δrrn* strains (Figure 4F); further, the levels of clustering in all strains exceed significantly the clustering seen in M9Glu. This result reinforces our qualitative observations of clustering in the discussion of the spatial RNAP distribution in RDM; the absence of prominent peaks in the projection of immobile RNAP localizations in the *Δrrn* strains is not due to the presence of highly distributed immobile RNAPs, but rather to the presence of RNAP clusters with variable positions along the long cell axis (due to the lack of synchronization of the cells, and, in turn, due to variable positions of the remaining *rrn* operons).

### The expected cellular *rrn* copy number in *Δrrn* strains can sustain the measured growth rates in M9Glu, but not in RDM

To evaluate whether the remaining *rrn* copies can accommodate the number of RNAPs needed to sustain the measured growth rate [which is proportional to both cellular ribosome content (hence to the rRNA cellular content) and peptide elongation rate (47)], we estimated the copy number of *rrn* operons (39) and of *rrn*-associated RNAPs (38) for the growth rates of our strains (Table 1), and used them to estimate the average fractional occupancy of each *rrn* operon (Table 1).

Due to the ongoing process of DNA replication in bacterial cells, and due to the uneven (and highly *ori*-proximal) distribution of the *rrn* operons on the chromosome, we expect for the WT strain (doubling time T of ∼47 min in M9Glu) an average of ∼2 genome equivalents, ∼17.5 *rrn* operons per cell (44) and ∼400 rRNAPs (40), yielding an average of ∼23 RNAPs/*rrn*. Considering a maximum *rrn* occupancy of ∼72 RNAPs [using the average *rrn* occupancy at the maximal *E. coli* growth rate of 24 min; (38)], the operons function at only ∼32% of their full capacity, hence being far from saturation and having significant spare capacity. We note that the value of ∼72 RNAPs/*rrn* is the RNAP occupancy at maximal growth rate (set by cell physiology), and not the maximal RNAP occupancy dictated by the physical RNAP footprint on the DNA (see the Discussion).

Regarding Δ5 (T ∼57 min), we expect to have ∼240 RNAPs engaged in *rrn* transcription and ∼5 *rrn* operons per cell, leading to an estimate of ∼50 RNAPs per operon, and ∼70% of max occupancy; even if we use our experimental result of ∼3.2 *rrn* foci/cell (see the last Results section), and assume conservatively that a focus contains only one *rrn* operon, we recover an upper bound that does not exceed the *rrn* operon capacity. These estimates strongly suggest that the remaining *rrn* genes in Δ5 can accommodate the number of RNAPs required for the observed growth rate. Similarly, even for Δ6, the doubling time of 73 min can be maintained by an ∼85% occupancy of the remaining ∼2 *rrnE* copies per cell. In essence, the *rrn* transcription requirements for the growth rates of the deletion strains in M9Glu can be fulfilled by relocating RNAPs to the remaining *rrn* operons. Regarding Δ7, which has a growth rate similar to that of the WT, the presence of ∼10 copies of the *rrn*-containing plasmid [pK4-16, based on pSC101, see also (28)] also provides enough *rrn* copies to sustain the required levels of rRNA.

A more complex picture emerges for the *Δrrn* strains in rich medium. In RDM, the WT (T ∼36 min) has ∼22 *rrn* operons per cell and ∼800 rRNAPs, with an average of ∼37 RNAPs/*rrn*, which corresponds to operating at ∼50% full capacity. As in the case of M9Glu, since we estimate that Δ7 has many copies (∼15) of the *rrn*B-containing plasmid, it can maintain high *rrn* transcription levels despite the loss of all chromosomal *rrn*; indeed, Δ7 shows the smallest fitness cost (increase in doubling time) amongst the *Δrrn* strains. However, the fact that Δ7 appears to show RNAP engagement that is closer to that of Δ5 and Δ6 (which have a lot fewer *rrn* copies) indicates that the RNAP association and/or transcription of *rrn* on the plasmid is not as effective as on the chromosome.

However, in the case of Δ5 (T ∼46 min, ∼420 RNAPs), we expect ∼5.6 *rrn* per cell based on the measured growth rate, hence ∼75 RNAPs/*rrn*, which exceeds the maximum capacity by ∼5%; this means that Δ5 is at the limit of being able to sustain growth by fully loading all remaining *rrn* operons with RNAPs. This limit is substantially exceeded in Δ6, the slowest growing strain in RDM (Τ ∼51 min), which requires ∼320 rRNAPs to maintain its growth rate, corresponding to ∼130 RNAPs/*rrn*.

This result strongly suggests that mechanisms other than simple RNAP relocation to the number of *rrn* copies expected purely on the basis of growth rate are needed to explain the ability of Δ6 (and, possibly, of Δ5) to sustain the observed growth rate in RDM.

### *rrn* transcription in rich medium for Δ6 is associated with reduced operon copy number per cell

Our estimates of RNAP occupancy of *rrn* in RDM clearly showed that, at least for the Δ6 strain, the cells cannot sustain the measured growth rate purely on the basis of the cellular number of *rrn* operons expected for the measured growth rate. To address this, we first tested the hypothesis that more *rrn* copies are generated due to increased replication initiation frequency.

**Figure 5.**
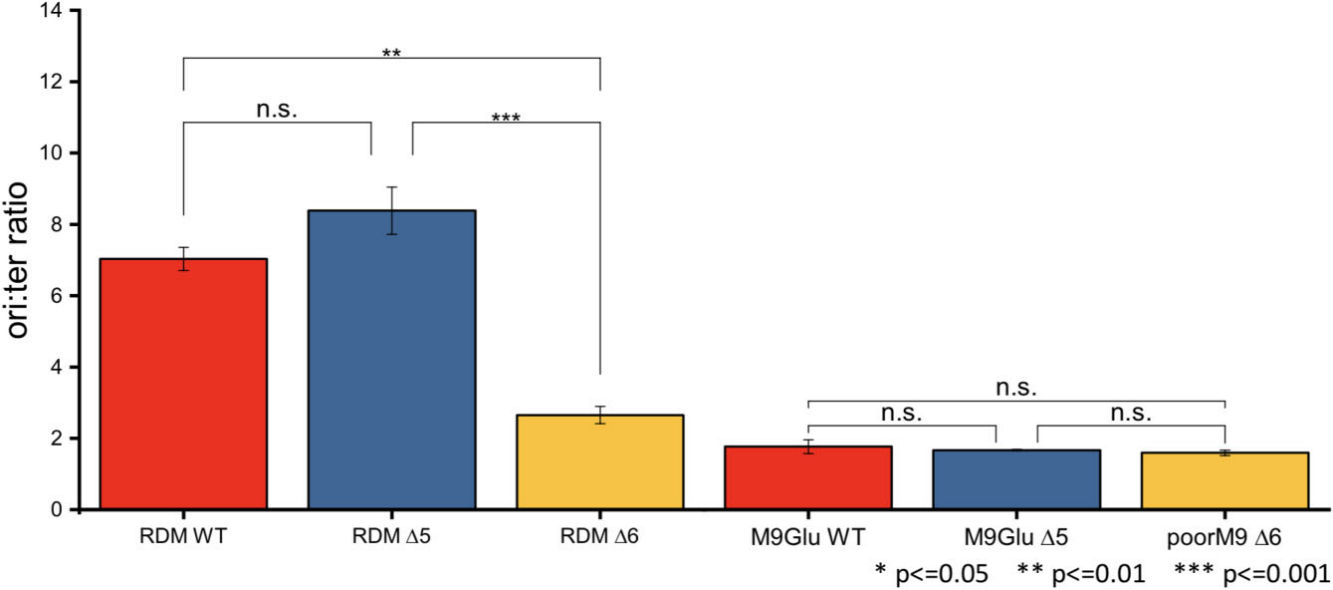
Measuring changes in replication initiation frequency. Measurements of the *ori:ter* ratio in a series of WT and Δ*rrn* strains in RDM and M9Glu media. The measurements were based on performing qPCR on genomic DNA (see the Materials and Methods). Error bars = SEM from three experimental repeats.

To examine whether the replication initiation frequency is affected in Δ6 relative to the WT, we performed quantitative PCR (qPCR) measurements of the *ori*:*ter* ratio, which acts as a proxy for the *rrnC*:*ter* ratio. The results (Figure 5A) clearly showed that the Δ6 strain grown in RDM shows a significantly lower *ori*:*ter* ratio compared with the WT (∼2.6 versus ∼7), and established that increased replication initiation cannot explain the high degree of RNAP engagement with the chromosome. The cell length distribution also showed that Δ6 cells are significantly shorter than those for the equivalent WT strain (∼2.8 versus ∼3.6 μm, respectively; Supplementary Figure S13).

The profile was very different for Δ5, which instead showed a small increase in the *ori:ter* ratio (from ∼7 to ∼8.4, albeit not statistically significant), while having a mean cell length similar to the WT strain (∼3.8 versus ∼3.6 μm; Supplementary Figure S2), and a slower growth rate than the WT. These results suggest some increase in replication initiation in Δ5, but any effect is fairly modest.

We also examined the WT, Δ6 and Δ5 strains in M9Glu (Figure 5), and found that they all had an *ori:ter* ratio of ∼1.5, suggesting that when the nucleoid number is small, loss of multiple *rrn* operons in the deletion strains does not lead to any significant changes in replication initiation, consistent with our expectations.

### Two-colour imaging reveals that RNAP clusters correspond to *rrn* foci

**Figure 6.**
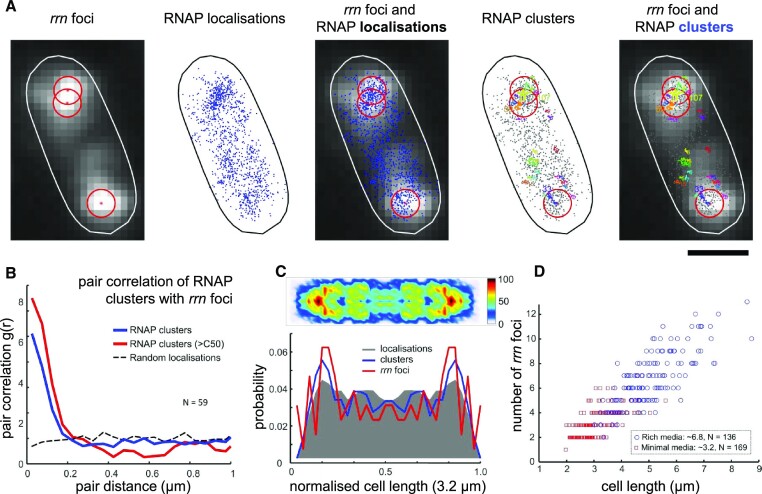
Co-localization of *rrn* foci with RNAP clusters in fixed cells. (**A**) Representative example of a fixed cell showing FISH-labelled *rrn* foci in Δ5 along with RNAP localizations and clusters. Each centroid corresponds to one *rrn* focus obtained by Gaussian fitting and marked by a red asterisk and a 200 nm radius red circle. RNAP localizations in fixed cells were analysed using DBSCAN to identify clusters; different clusters, along with their number of RNAPs, are shown in different colours. Scale bar, 1 μm. (**B**) Pair correlation analysis of *rrn* foci with all RNAP clusters, as well as large RNAP clusters (C > 50) according to the pairwise distance show high co-localization between *rrn* foci with RNAP clusters and C > 50 clusters: ∼46% of RNAP clusters and ∼77% of RNAP large clusters locate within a 200 nm radius of *rrn* foci (blue line), compared with ∼23% obtained for simulated localizations randomly distributed throughout the nucleoid area (dashed black line). (**C**) Heatmap of RNAP clusters and their projections (blue line). Projections of localizations and *rrn* foci are displayed in a grey shaded area and red line, respectively. (**D**) Scatter plot of *rrn* foci number in Δ5 in M9Glu and RDM as a function of cell length. The mean number of *rrn* foci is 3.2 ± 0.1 (SEM) in M9Glu, and 6.8 ± 0.2 (SEM) in RDM.

To provide direct evidence for the link between RNAP clusters and *rrn* operons in the *Δrrn* strains, we performed two-colour co-localization assays by combining FISH imaging of *rrn* foci with single-molecule RNAP localization in Δ5. Similarly to published work (13), which showed that RNAP clusters co-localize with nascent rRNA in WT cells grown in rich medium, we used a fluorescent FISH probe that targeted the 5′ leader region of the 16S precursor rRNA (pre-rRNA), which is absent from mature rRNA and ribosomes (see the Materials and Methods). Signal from our FISH probe in fixed cells in M9Glu allows us to capture transcribing *rrn* foci, visualized as bright diffraction-limited spots in pole-proximal regions (Figure 6A). When used in conjunction with PALM data, the relative co-localization identified active *rrn* clusters in Δ5 and estimated their copy numbers in M9Glu and RDM to be ∼3.2 ± 0.1 and ∼6.8 ± 0.2, respectively (Figure 6D). To record the position of *rrn* foci, its centroid was determined by a Gaussian fitting and superimposed on RNAP localizations and RNAP clusters; we observed that most RNAP clusters (especially large clusters containing > 50 localizations; C > 50) locate within 200 nm from the *rrn* centroid (Figure 6A), suggesting significant co-localization between RNAP clusters and *rrn* foci.

To quantify the degree of co-localization, we performed pair correlation analysis between the *rrn* loci and RNAP clusters (either using all clusters with N ≥ 4, or large C > 50 clusters). Our results show a high correlation between *rrn* foci and RNAP clusters, supporting their co-localization. Specifically, we found that ∼46% of all RNAP clusters, and ∼77% of RNAP large clusters localize within 200 nm of *rrn* foci, compared with ∼23% expected on the basis of simulated random RNAP localizations (Figure 6B), which employs similar analysis algorithms to our pair correlation analysis in Figure 4.

To visualize the distribution of RNAP clusters, we generated the heatmap of RNAP clusters from the normalized positions of clustered RNAPs in Δ5 grown in Μ9Glu (Figure 6C). Our results clearly show that RNAP clusters are concentrated at pole-proximal regions, a fact also reflected in projections along the cell length axis. The projection of the normalized positions of *rrn* foci also display pole-proximal peaks, which highly overlap with the peaks of RNAP clusters (Figure 6C). These results clearly establish the physical proximity of the RNAP clusters and *rrn* foci, and further support the suggestion that RNAPs relocate to the remaining *rrn* operons to sustain high levels of *rrn* transcription and largely maintain the growth rate achieved in the absence of any *rrn* deletions.

## DISCUSSION

The spatial organization of RNAP in bacteria has been a long-standing question ever since the first observations of transcription foci in cells grown in rich medium (1–3,8,48–50), and the linkage between transcription foci and rRNA synthesis has remained controversial (1). Here, we applied super-resolution imaging and single-molecule tracking on strains with a heavily reduced number of *rrn* operons to elucidate the relationship between RNAP spatial organization and rRNA synthesis, and study how cells redeploy their transcription machinery to sustain a healthy growth rate with only one or two chromosomal *rrn* operons. Notably, most bacterial species (∼80%) have 1–4 *rrn* copies in their genome, with ∼35% having just 1–2 copies (51); a large number of *rrn* copies enables the provision of high numbers of ribosomes per cell, which in turn allows bacteria harbouring a large *rrn* number to adapt more quickly to nutritional upshifts and switch to fast growth (and, in general, respond more rapidly to changes in the nutrient availability) (51).

### RNAPs maintain their chromosome engagement in *Δrrn* strains by increasing the loading of the remaining *rrn* operons

Our RNAP mobility analysis showed that the fraction of immobile RNAPs, a proxy for the fraction of RNAPs engaged in transcription (plus any RNAPs involved in condensates), is surprisingly robust to the loss of five and six chromosomal *rrn* copies, as well as to the loss of all chromosomal *rrn* copies when cells are supplemented by a low copy-number plasmid harbouring a single *rrn* operon. In M9Glu, a medium that supports a doubling time of 47 min in the WT, about half of all RNAPs were immobile both for the WT [as seen in (12,52)] and for all *Δrrn* strains. Even in rich medium (RDM; supporting a doubling time of 36 min in the WT), heavy loss of *rrn* operons led to only a modest decrease in the immobile RNAP fraction (63% for the WT; 57–58% for the *Δrrn* strains).

**Figure 7.**
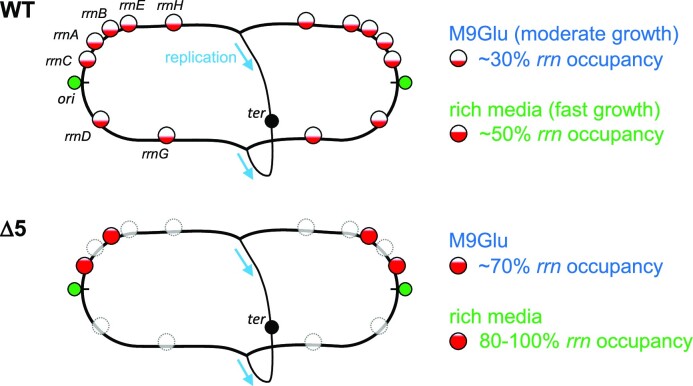
RNAP redistribution maximizes *rrn* transcription and minimizes growth defects in *E. coli* strains with a small number of *rrn* operons. The *E. coli* strain with all seven chromosomal *rrn* copies carries a large number of *rrn* copies (>15 copies) per cell either in M9Glu or in rich medium, with the operons being far from saturation (30–50% of the occupancy seen in cells growing at a maximal rate) in both media, and RNAP clusters forming on *rrn* spreading throughout the entire nucleoid. In contrast, in *rrn* deletion mutants lacking most *rrn* copies from the chromosome, the remaining *rrn* copies are much more occupied by RNAPs (∼70% in Δ5 in M9Glu), and RNAP clusters are more concentrated in the pole-proximal positions. More demanding growth conditions with regard to rRNA resources, such as in rich medium, maximize the RNAP occupancy on the remaining *rrn* operons, while they also increase the transcription of some mRNA genes that deal with the consequences of replication–transcription conflicts in heavily transcribed *rrn* operons, as manifested in the Δ6 strain grown in rich medium.

The robustness of the RNAP immobile fraction to the loss of most chromosomal *rrn* genes raises the question of how RNAPs ‘released’ from the deleted *rrn* operons redistribute to other immobile fractions, and how this redistribution minimizes any growth rate defects in *Δrrn* strains. Under our growth conditions in the WT strain (Figure 7, top), we expect that *rrn* promoters are not saturated with RNAP; as a result, an increase in the concentration of available free RNAP should lead to increased *rrn* promoter activities (47). Our results support a scenario where the remaining *rrn* copies in the cell are more heavily loaded by RNAPs (Figure 7, middle).

Such RNAP re-distribution had been observed in a study of a more limited *rrn* deletion in rich LB medium; specifically, deletion of four *rrn* operons (Δ4) resulted in the remaining *rrn* operons accommodating ∼71 RNAPs, an increase from 53 RNAPs/*rrn* in the strain with all operons intact, with the increased occupancy being linked to increased transcription initiation and elongation (40). Notably, the growth slow-down seen in Δ4 (∼24 min for WT versus ∼30 min for Δ4) is similar to that we see for Δ5 in RDM.

Our observations are consistent with the ‘saturation model’ for the passive regulation of gene expression (47,53), and with studies showing that a 2- to 3-fold overexpression of the RNAP-σ70 holoenzyme leads to a 2-fold increase of *rrnB*P1 transcription, and a large increase in the tendency to transcribe *rrn* genes versus mRNA genes (54). In general, our results clearly show that the *rrn* promoters are competing very effectively against mRNA promoters.

### RNAP clusters do form on *rrn* operons

Our results clearly establish that RNAPs are more clustered in the *Δrrn* strains, and that RNAP clusters are in physical proximity to the *rrn* foci, adding further support to the proposal that RNAPs relocate to the remaining *rrn* operons to sustain high levels of *rrn* transcription and largely maintain the growth rate achieved in the absence of any *rrn* deletions. Our results are consistent with results from Weng *et al.* (13), where it was shown that large RNAP clusters are maintained in a strain with a single *rrn* on the chromosome in rich medium, as well as during low levels of transcription (13).

The presence of clustering on *rrn* (rRNAPs) does not exclude the presence of other forms of clustering, such as condensates (cRNAP) or heavy transcription on mRNA genes. It demonstrates, however, the ability of RNAP to redistribute and reprogram gene expression due to the cellular response to changes in the local environment or chromosome context (1,13).

### The location of remaining *rrn* operons dictates the location of the clusters in M9Glu

Considering the genomic map (Figure 1B), the remaining *rrn* operons in the deletion mutants are either near *ori* (40), which is situated at pole-proximal regions along the cell long axis (45), or on plasmids known to localize preferentially at the polar endcaps; these positions are consistent with the new peak of localizations (along the long cell axis) that appears in all three *Δrrn* strains (Figure 2D). Relocation of released immobile RNAPs in M9Glu in *Δrrn* to pole-proximal positions is thus dictated by the places of the remaining *rrn* operons.

The presence of a well-defined location for the relocated rRNAPs in the case of M9Glu makes it unlikely that the released rRNAPs relocate to transcribe mRNA, since such a relocation would have resulted in a much more evenly distributed spatial profile for RNAP. Equally, our results are not consistent with rRNAP relocation to any RNAP-containing condensates not associated with transcription, since it is unlikely that such condensates will have the same spatial patterns as those dictated by the locations of the remaining chromosomal *rrn* and the plasmid-borne *rrn* operons. In essence, our results suggest that it is the clustered rRNAPs that recruit cRNAPs (at least in the M9Glu case), and not the other way around.

### Heavy loss of *rrn* operons in rich medium leads to RNAP redistribution to both rRNA and mRNA genes

Our measurements of the *ori:ter* ratio indicated that in Δ6 in rich medium, mechanisms additional to RNAP relocation to the remaining *rrn* operons are necessary, since the number of *rrn* operons is reduced by ∼3 fold. The reason for this decrease is likely to be high levels of blocked DNA replication and high DNA damage due to transcription–replication conflicts in the remaining *rrn* operon, as shown in a strain similar to Δ6 (55); the same work also showed that such a strain was linked with induction of the SOS response (activation of ∼40 genes). Given our results and the work of Fleurier *et al.* (55), it is highly likely that Δ6 cells in RDM are in a stressed/DNA-damaged state, and, in this state, they re-distribute many RNAP molecules to non-*rrn* genes, in order to balance a moderate growth with the need to minimize DNA damage and repair their DNA.

Our initial estimates predicted that ∼320 RNAPs were needed to produce the ∼20 000 ribosomes needed to sustain the growth rate of Δ6 (39); this number of rRNAPs corresponds to ∼128 RNAPs per *rrn* (given the expected number of *rrn* copies for the Δ6 growth rate; see Table 1). This number exceeds substantially the number of ∼72 RNAP/*rrn* observed at maximal growth and constrained by cell physiology. However, the maximal physical RNAP occupancy is considerably higher. The footprint of elongating RNAP on DNA is only 25–35 bp [as determined by DNase I footprinting (56)]; further, the amount of DNA covered by RNAP in structures of elongation complexes is ∼30 bp (57). If we instead use a footprint of 40 bp per elongating RNAP, this will yield a maximal physical occupancy of 135 RNAPs/*rrn*, which would in principle be able to support the Δ6 growth rate in RDM. However, such a dense occupancy is clearly toxic for cells, since it leads to substantial DNA damage (55). This also means that the maximal growth rate RNAP occupancy is kept substantially lower than the physical one to avoid problems with genomic instability.

Notably, the profile in rich medium is very different for the Δ5 strain, which presumably avoids the high levels of DNA damage seen in Δ6 by having two chromosomal *rrn* copies and benefiting from high gene dosage (*ori:ter* ratio of 8.4 and 1.5 for Δ5 in RDM and Μ9Glu, respectively). Since the *rrn* operons will be close to full occupancy in RDM, we also speculate that they will be associated with co-directional transcription–replication conflicts and increased replication restart, as has been reported previously for highly transcribed *rrn* operons in *B. subtilis* (58).

## Supplementary Material

gkad511_Supplemental_File

## Data Availability

Movies and images of cells as well as localization files for single molecules will be available upon request.
