# Supplementary material for: RNA polymerase redistribution supports growth in E. coli strains with a minimal number of rRNA operons
Source: Nucleic Acids Res. 2023 Jun 23;51(15):8085–101. doi: 10.1093/nar/gkad511 (PMC10450203; doi:10.1093/nar/gkad511)
Supplement: gkad511_Supplemental_File [file gkad511_supplemental_file.pdf]

Supplemental Figures for Fan *et al* manuscript

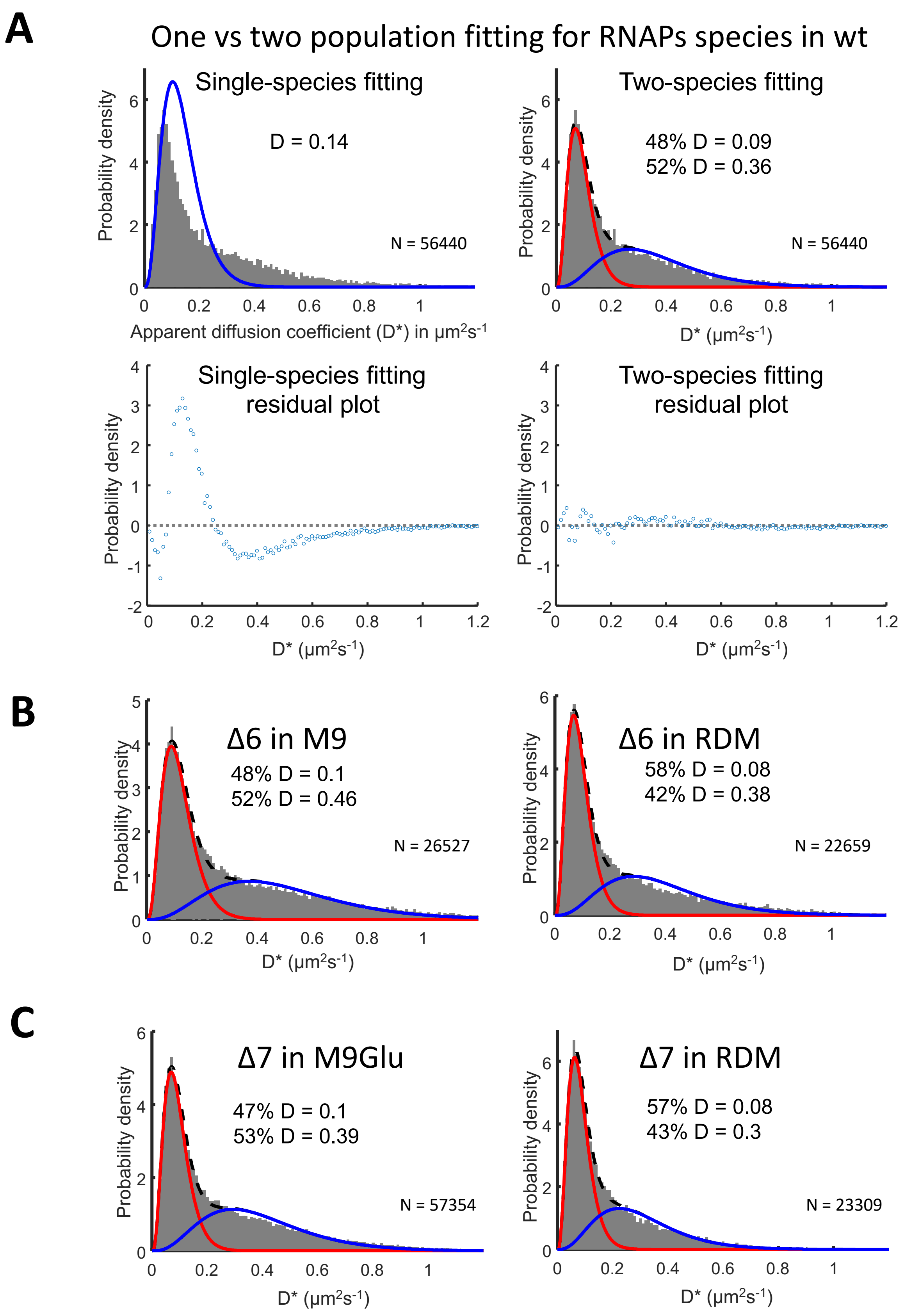

SuppFig. 1. Bound RNAP fraction in deletion mutants. A. Fittings with single- or two-species for histogram of apparent diffusion coefficient (D*) in wild type with M9Glu media. The residual plots are below the fitting panels. **B.** Histograms of D* fitted with two-gamma distributions for Δ6 in the M9Glu (left) and rich media (right). The mean fractions of bound and mobile RNAPs are listed. **C.** Histograms of D* fitted with two-gamma distributions for Δ7 in the M9Glu (left) and rich media (right). The mean fractions of bound and mobile RNAPs are listed. Bar plots of the fractions in all the strains are shown in Fig1.

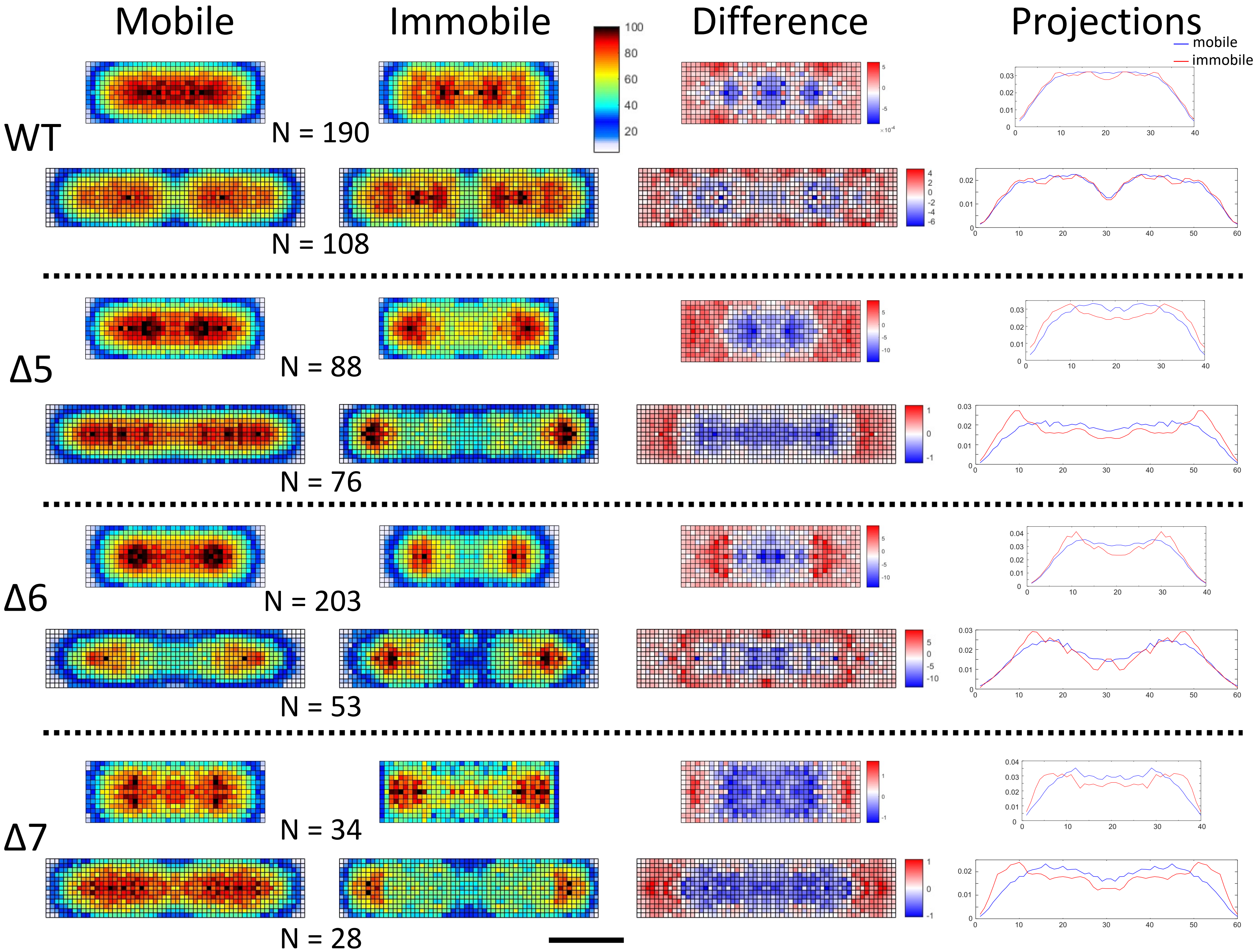

Fig. S2. Heatmaps of mobile and immobile RNAPs and projections along the long-axis for all strains in M9Glu media. For each strain, cells were sorted into a short group (top: 1.8-3 µm) and long group (bottom: 3.2-4 µm). In all *Δrrn* strains in M9Glu, immobile RNAPs redistribute to subcellular regions in close-proximity to the pole. Mobile RNAPs distribute throughout the nucleoid for all strains. The redistribution of immobile RNAPs is also reflected in the difference map (calculated by subtraction of mobile RNAPs from immobile RNAPs) and projection curves along the long axis of the cells. Scale bar, 1 µm.

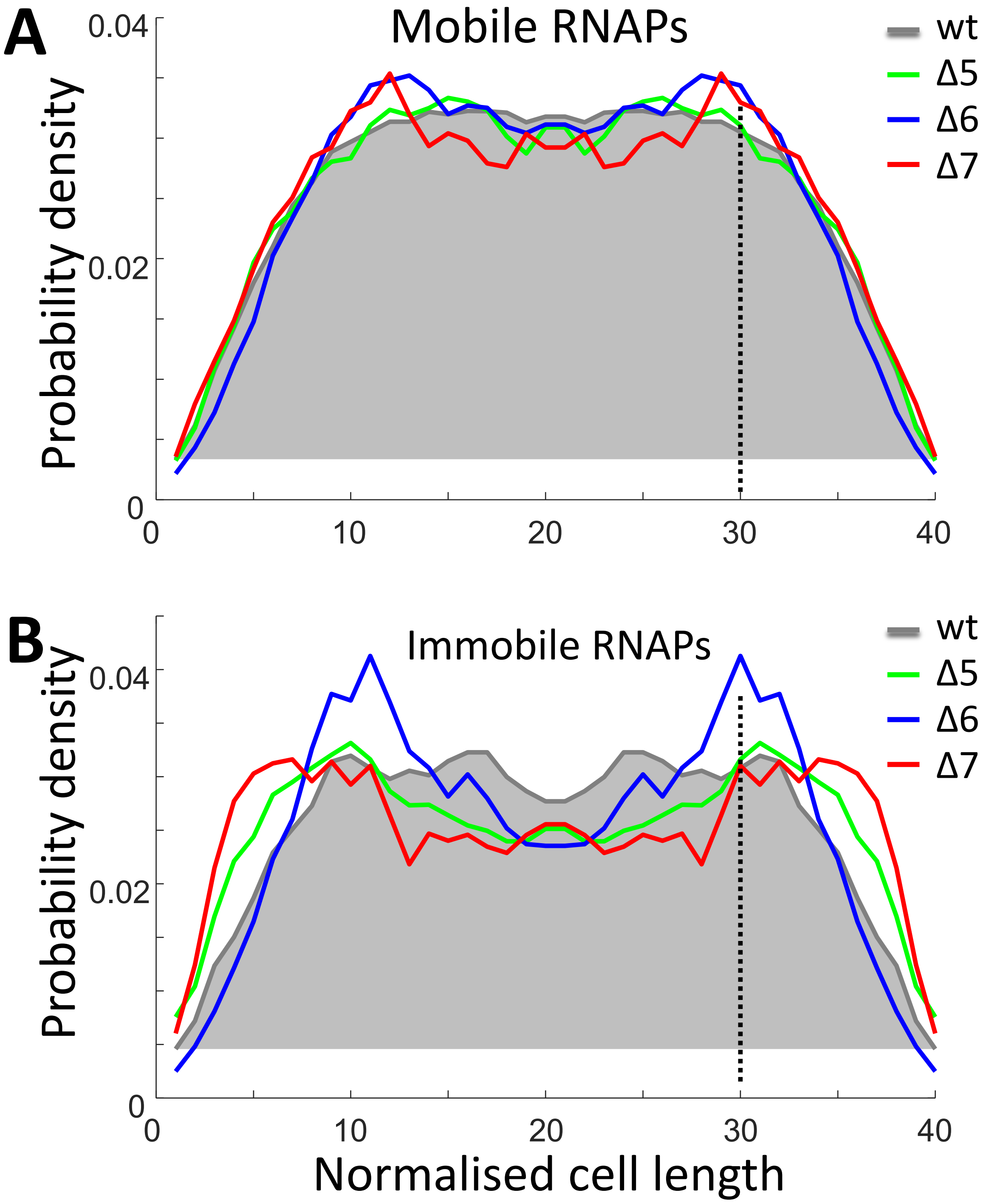

C

Strains	Exterior fractions	
	Mobile	Immobile
wt	37%	39%
Δ5	37%	47%
Δ6	34%	40%
Δ7	39%	50%

Fig. S3. Spatial distributions of the mobile and immobile RNAPs for short cells in M9Glu media. A-B. Projections of mobile (**A**) and immobile (**B**) RNAPs along the long axis of the heatmaps for the short-cell group (1.8-3 µm length range) in M9Glu. WT projection is shown as a gray-shaded area. Dashed vertical lines indicate the 25% position along the long axis. **C**. Fractions of RNAPs localised in the exterior 25% region along the long axis.

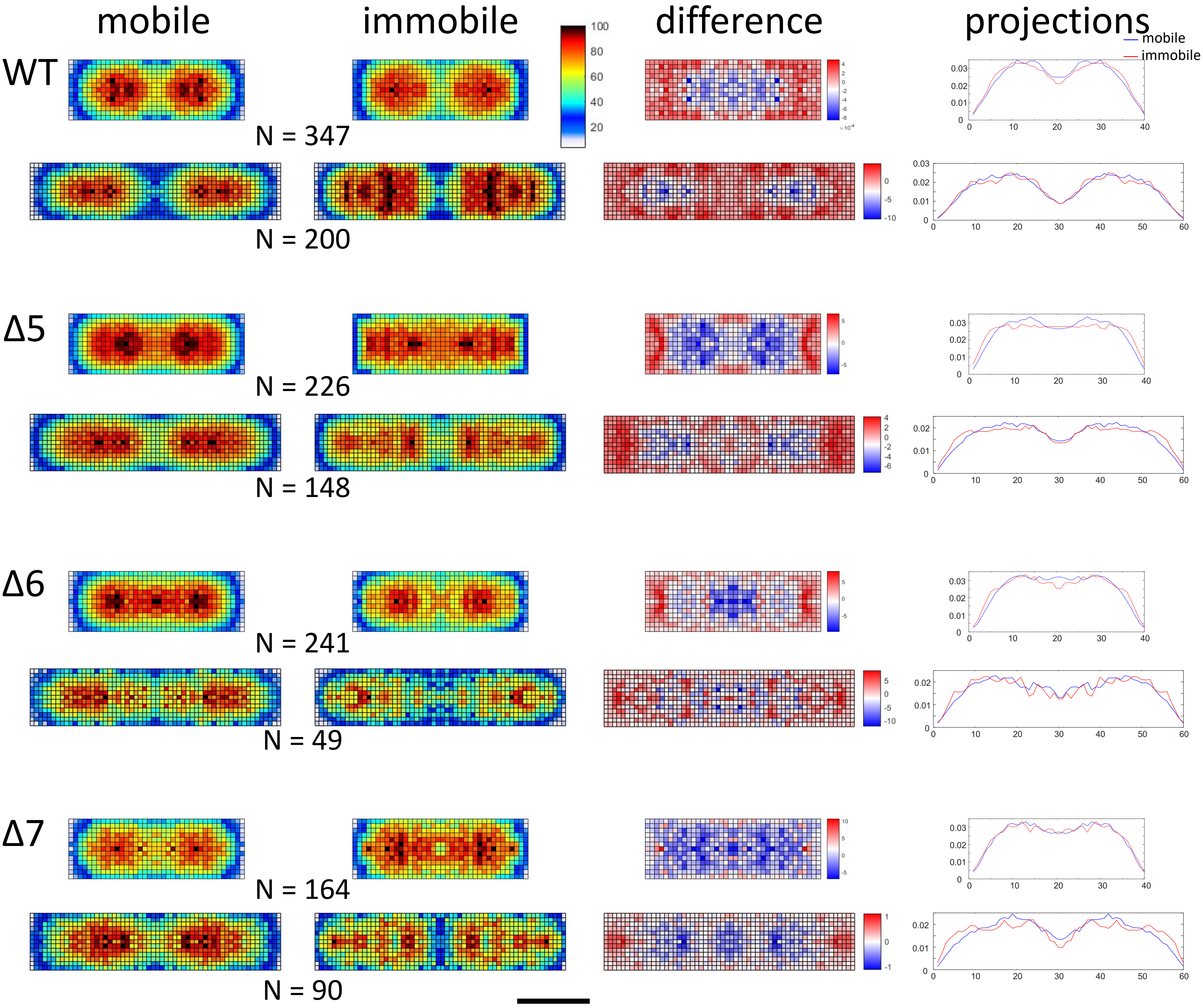

Fig. S4. Heatmaps of mobile and immobile RNAPs and projections along the long axis for all strains in rich media. For each strain, cells were sorted into short group (top: 1.8-3.5μm) and long group (bottom: 3.5-4.5μm). Scale bar, 1 μm.

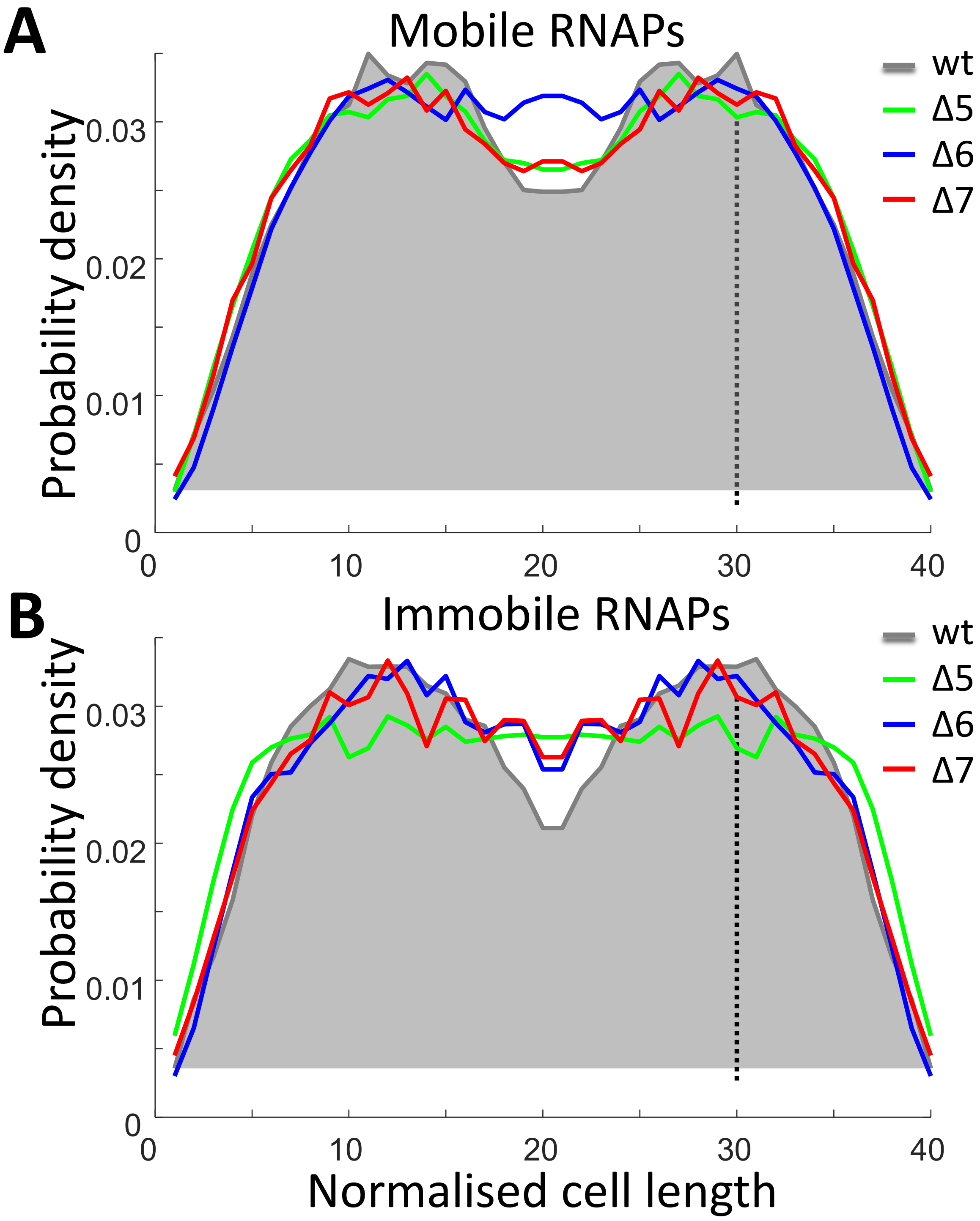

C

Strains	Exterior fractions	
	Mobile	Immobile
wt	38%	42%
Δ5	40%	44%
Δ6	37%	40%
Δ7	40%	41%

Fig. S5. Spatial distributions of the mobile and immobile RNAPs for short cells in RDM media. A-B. Projections of mobile (**A**) and immobile (**B**) RNAPs along the long axis of the heatmaps for the short-cell group (1.8-3.5 μm length range) in RDM. WT projection is shown as a gray-shaded area. Dashed vertical lines indicate the 25% position along the long axis. **C**. Fractions of RNAPs localised in the exterior 25% region along the long axis.

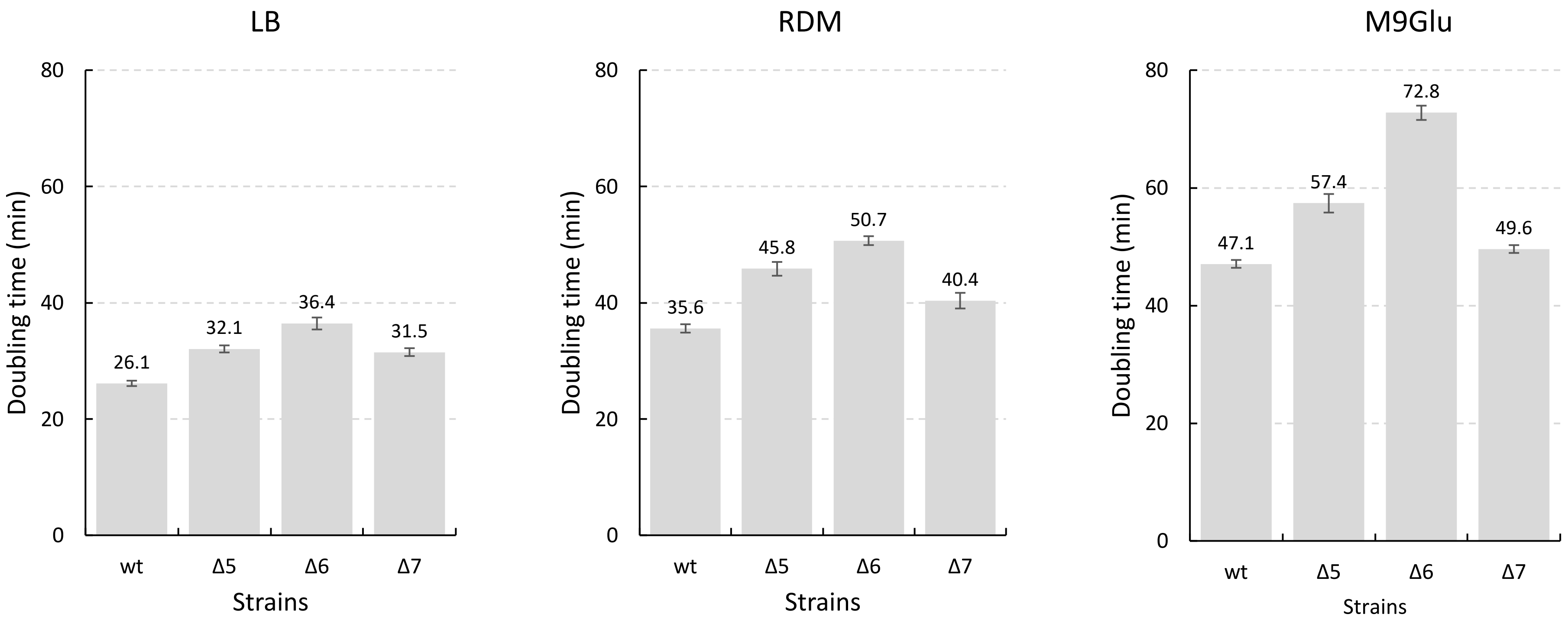

Fig. S6. Doubling times of wild type and *Δrrn* strains growing at 37°C in different growth media. Left: LB. Middle: RDM. Right: M9Glu. The means of the doubling times are presented on top of each column; error bars reflect for standard error of the mean (SEM).

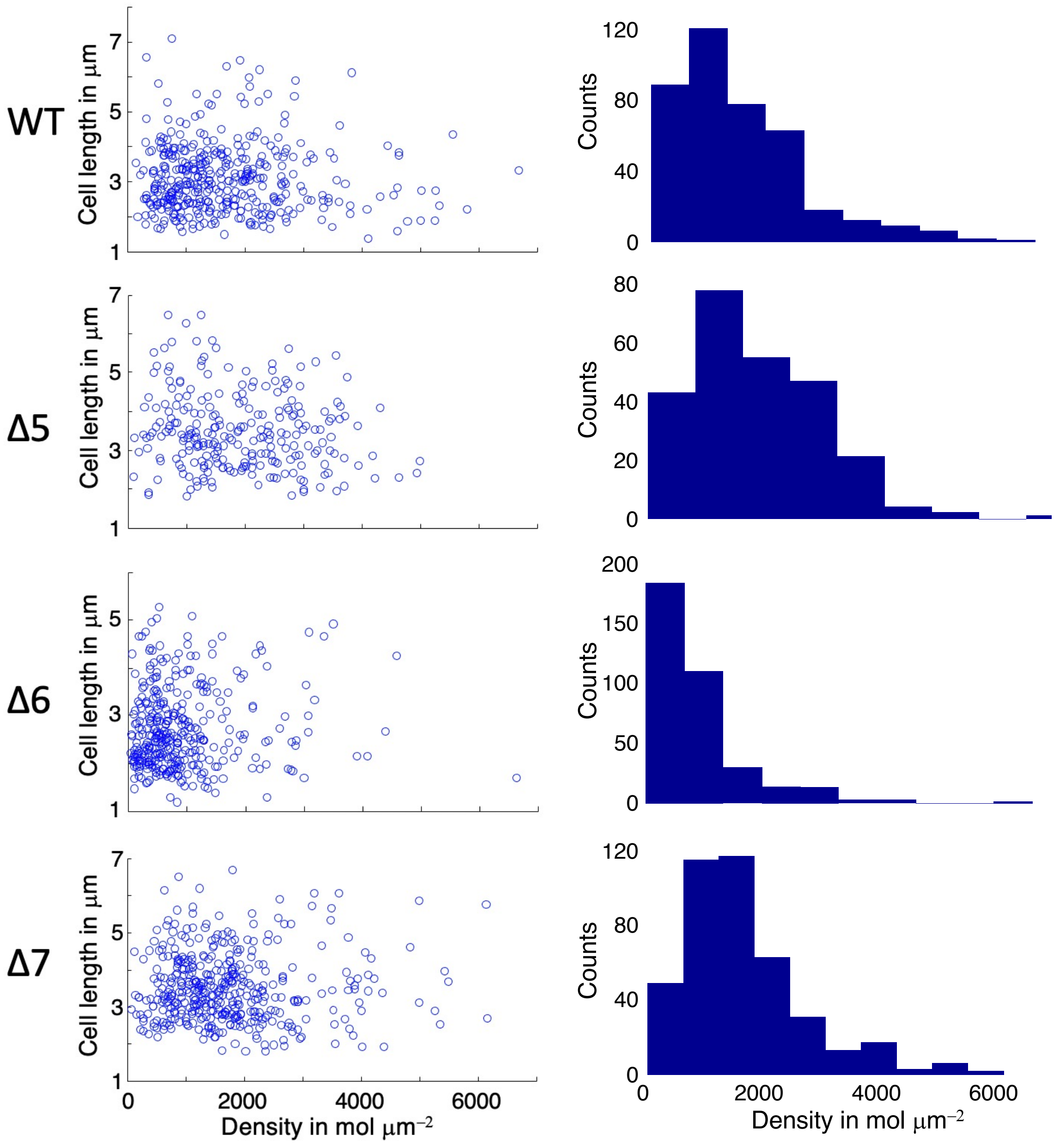

Fig. S7. Acquired RNAP localizations for all strains in M9Glu media. Left side: scatter plot of localizations as a function of cell length. Right side: histograms of localisation density.

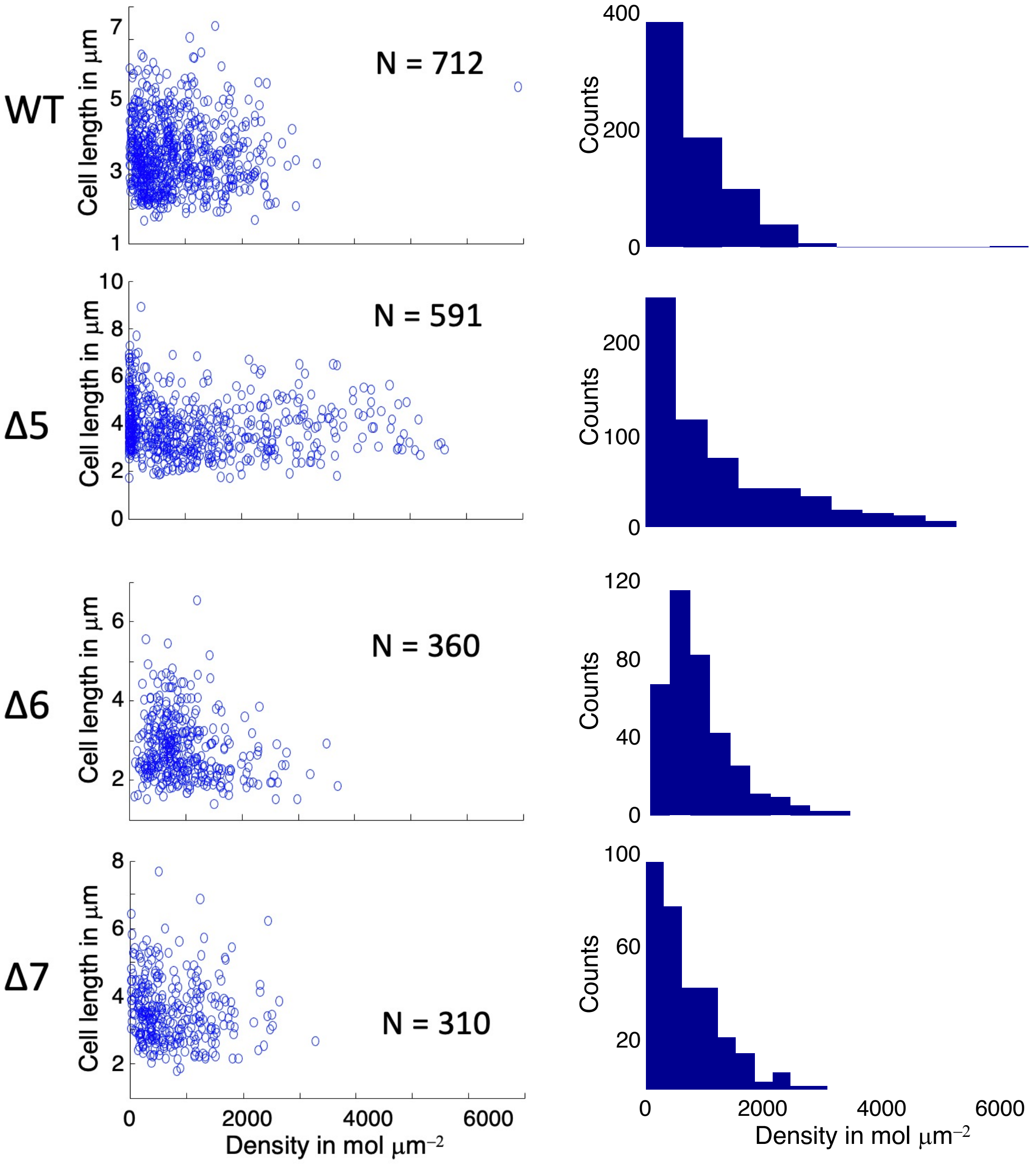

Fig. S8. Acquired RNAP localizations for all strains in RDM media. Left side: scatter plot of localizations as a function of cell length. Right side: histograms of localisation density. N denotes number of cells.

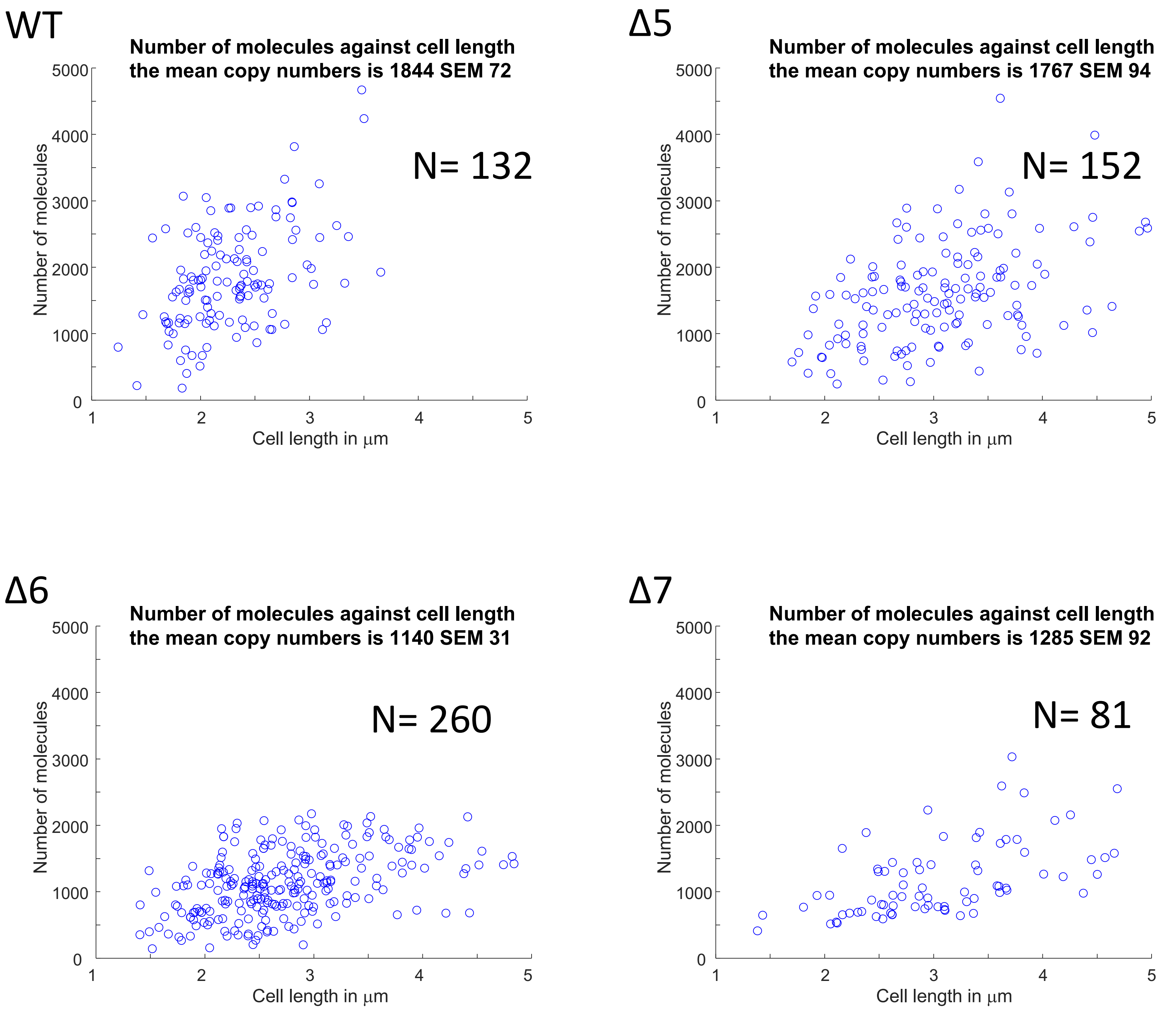

Fig. S9. RNAP copy number measurement based on PALM measurements in fixed cells in M9Glu media. The mean copy number and SEM per strain is shown as in the title of each panel. N denotes number of cells.

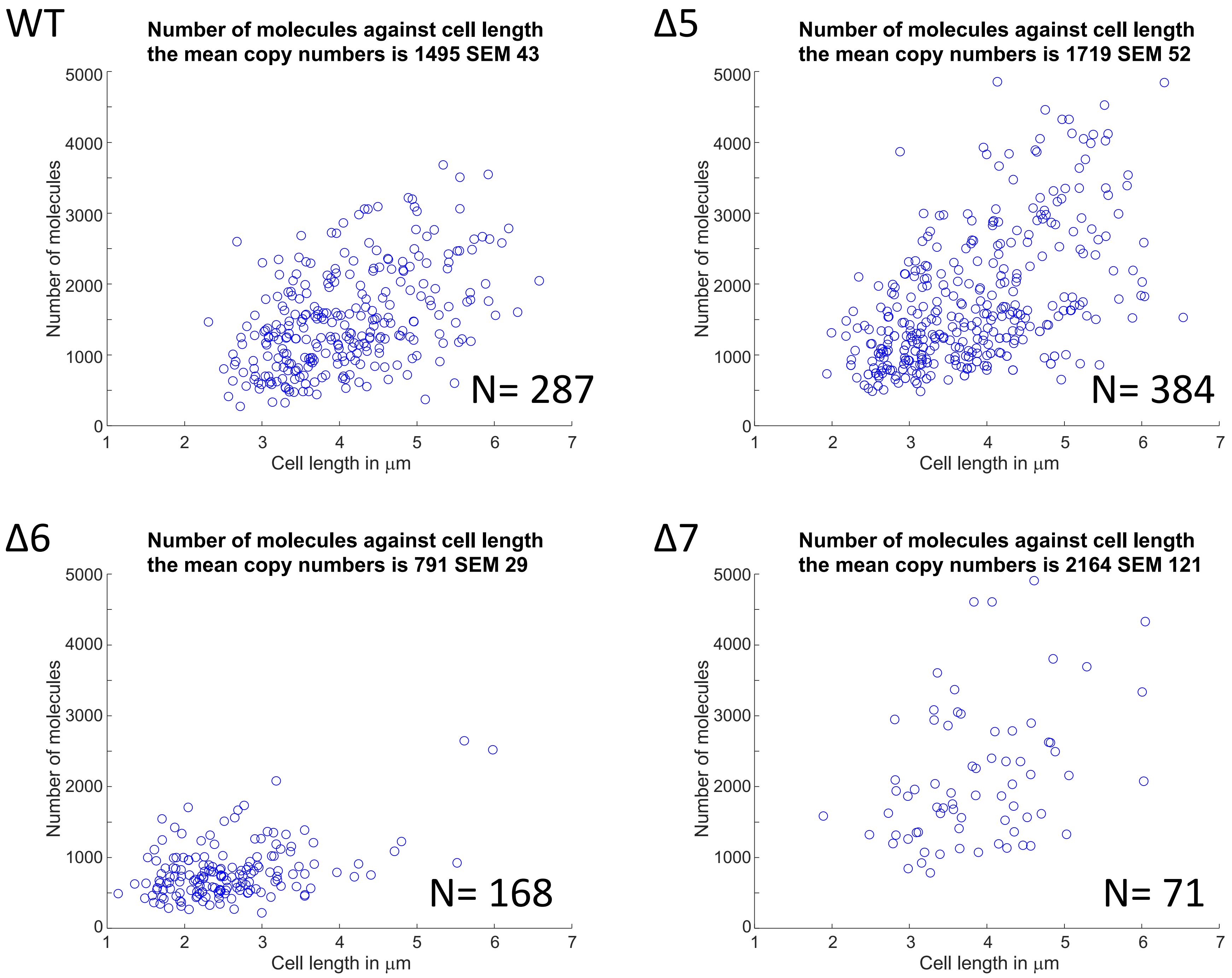

Fig. S10. RNAP copy number measurement based on PALM measurements in fixed cells in RDM media. The mean copy number and SEM per strain is shown as in the title of each panel. N denotes number of cells.

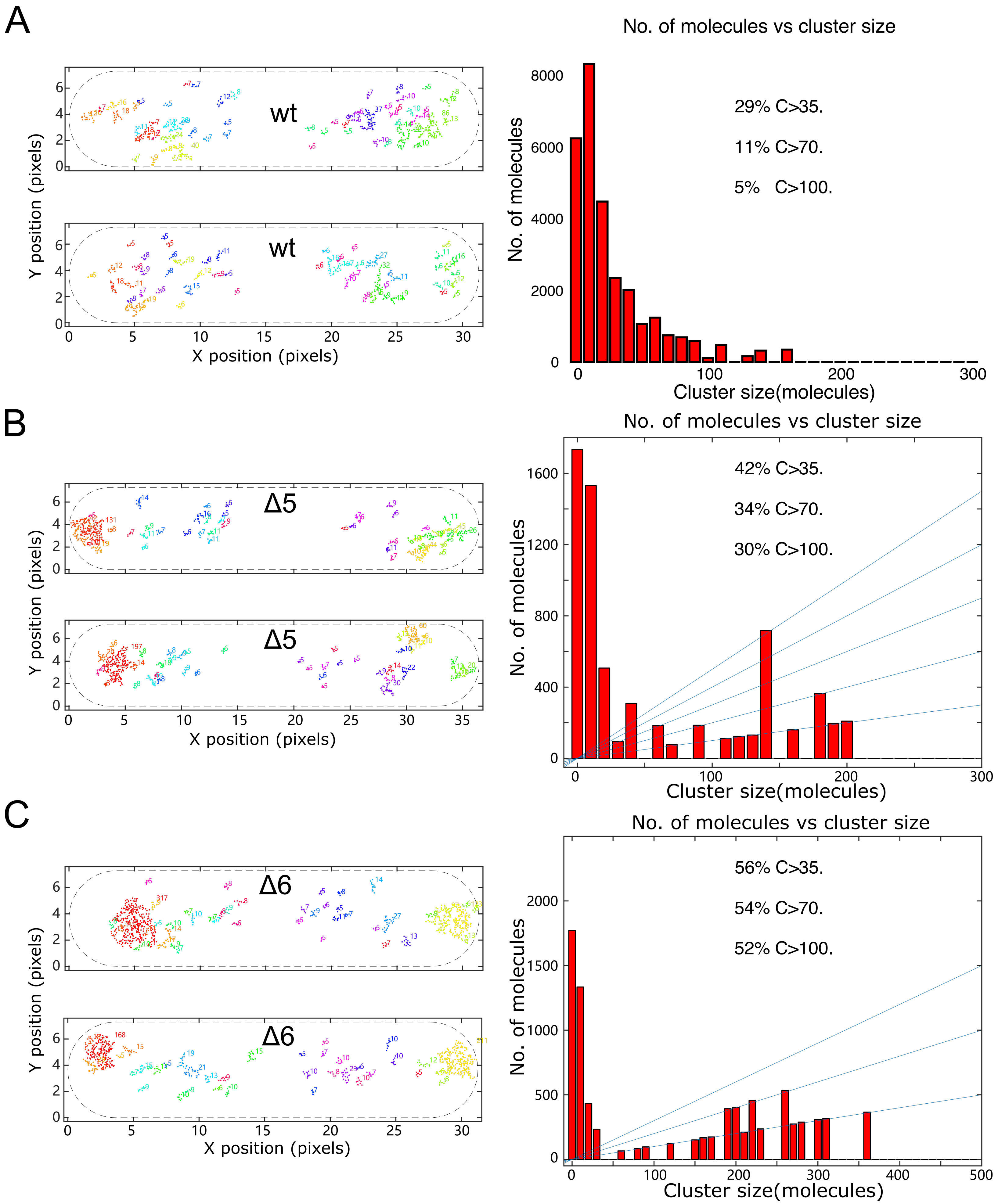

Fig. S11. Representative cell examples with RNAP clusters by simulation for wt, Δ5 and Δ6 and related clusters histograms. For a detailed description, see *Methods.* **A-C.** Simulated RNAP localisations (left columns) and frequency histogram for identified RNAP clusters of different size (right columns) in WT cells (panel A), Δ5 (panel B) and Δ6 (panel C).

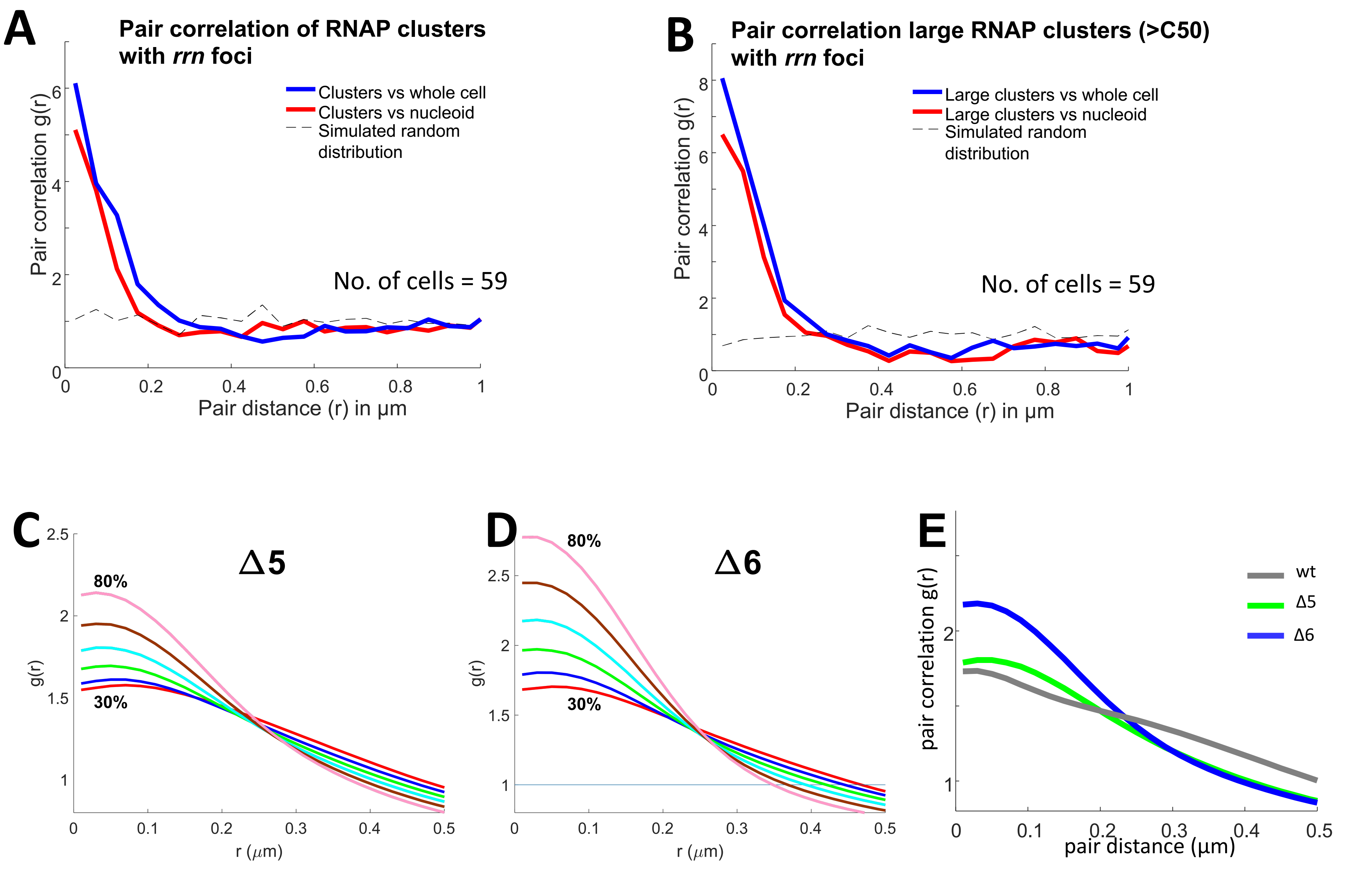

Fig. S12. Pair correlation of RNAP clusters with *rrn* foci and simulated data for Δ5 and Δ6. A. Pair correlation of RNAP clusters in Δ5 in M9Glu normalised with whole cell (blue curve) and with nucleoid (red curve), compared with simulated localisations throughout the nucleoid. The fraction of clustered RNAPs within 200nm of *rrn* foci: 46.2 ± 2.5 % (SEM) and the fraction of random localisations throughout the nucleoid within 200nm of *rrn* foci 22 ± 1.6 %(SEM). **B.** Pair correlation of large RNAPs clusters (C>50) in Δ5 in M9Glu media normalised with whole cell (blue curve) and with nucleoid (red curve), compared with simulated localisations throughout the nucleoid. The fraction of heavily-clustered RNAPs (>C50) within 200nm of *rrn* foci: 77.3 ± 5.2 % (SEM) and the fraction of random localisations throughout the nucleoid within 200 nm of *rrn* foci 23 ± 1.9 % (SEM). **C-D.** Pair correlation of RNAPs by simulated data for Δ5 and Δ6. Percentages indicate the fractions of immobile RNAPs covered by individual *rrn*. As coverage increases from 30% to 80%, RNAPs pair correlation levels increase. **E**. Pair-correlation analysis of simulated RNAP localisations for WT and two *Δrrn* strains in M9Glu.

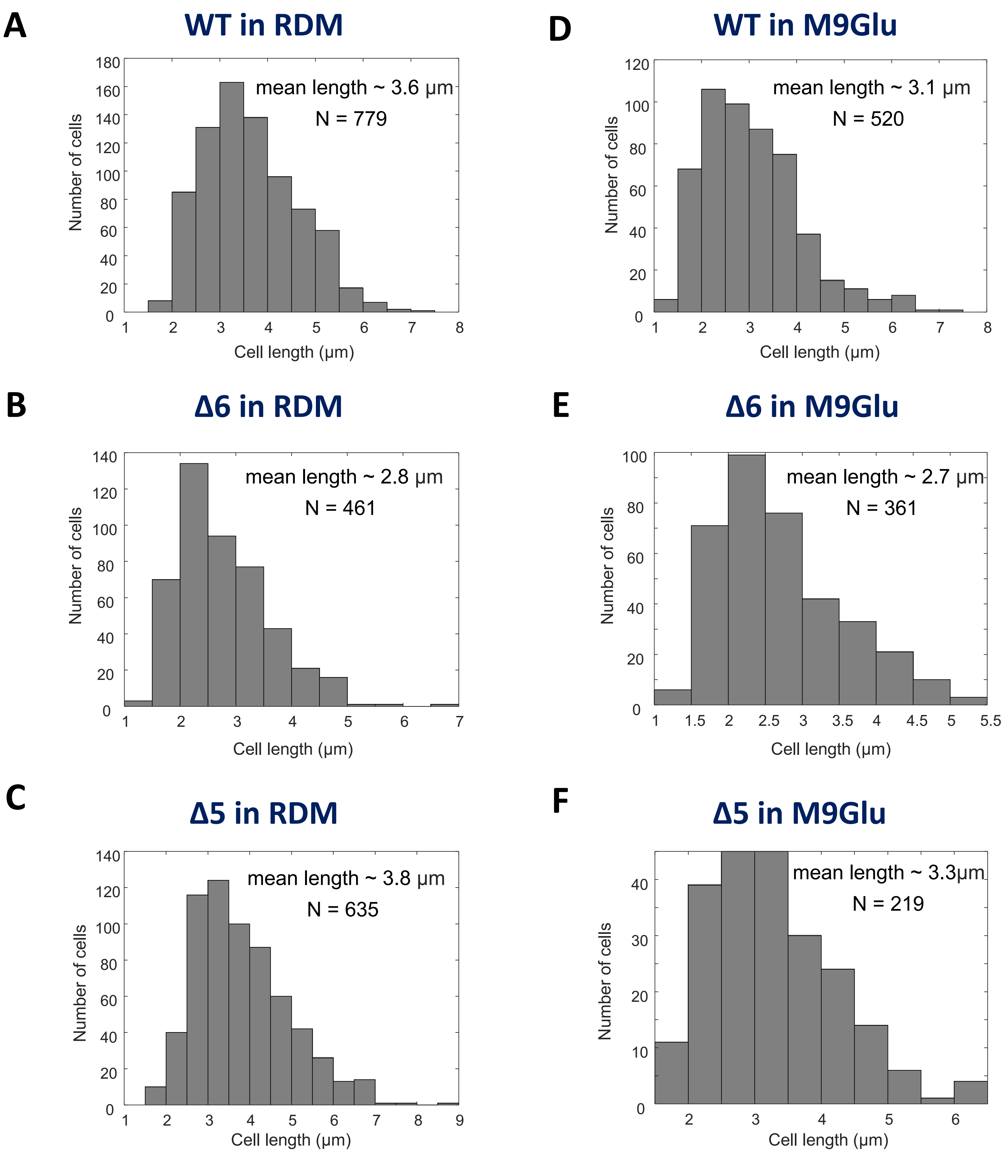

Fig. S13. Cell-length distributions for WT, Δ6 and Δ5 strains grown in RDM (panels A-C) and M9Glu (panels D-F). *N*, number of cells.
